# Anomalous inapplicability of nacre-like architectures as impact-resistant templates in a wide range of impact velocities

**DOI:** 10.1038/s41467-022-35439-3

**Published:** 2022-12-13

**Authors:** Xiao Zhang, Kaijin Wu, Yong Ni, Linghui He

**Affiliations:** CAS Key Laboratory of Mechanical Behavior and Design of Materials, Department of Modern Mechanics, CAS Center for Excellence in Complex System Mechanics, University of Science and Technology of China, Hefei, Anhui 230026 China

**Keywords:** Bioinspired materials, Bioinspired materials, Mechanical properties

## Abstract

Nacre is generally regarded as tough body armor, but it was often smashed by predators with a certain striking speed. Nacre-like architectures have been demonstrated to dissipate abundant energy by tablets sliding at static or specific low-speed loads, but whether they’re still impact-resistant templates in a wide range of impact velocities remains unclear. Here, we find an anomalous phenomenon that nacre-like structures show superior energy-dissipation ability only in a narrow range of low impact velocities, while they exhibit lower impact resistance than laminated structures when impact velocity exceeds a critical value. This is because the tablets sliding in nacre-like structure occurs earlier and wider at low impact velocities, while it becomes localized at excessive impact velocities. Such anomalous phenomenon remains under different structural sizes and boundary conditions. It further inspires us to propose a hybrid architecture design strategy that achieves optimal impact resistance in a wide range of impact velocities.

## Abstract (web summary)

Nacre structure is used as inspiration in the design of impact resistant materials yet natural nacre is overcome by high impact speed attacks from predators. Here, the authors perform a range of testing and demonstrate superior energy dissipation of nacre-like structures at low impact velocities which is lost at higher impact velocities.

## Introduction

**Fig. 1:**
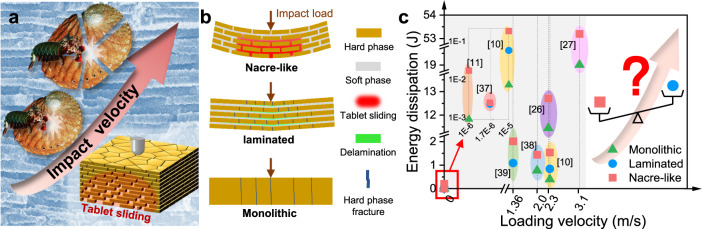
The velocity-dependent impact resistance of nacre-like structures. **a** The schematic illustration of nacre from abalone shells smashed by predators with the increase of the impact velocity, where tablet sliding over large volume is key to impact-energy dissipation. **b** The schematic of energy-dissipation mechanisms of the nacre-like structure, laminated structure and monolithic structure under impact. **c** The energy dissipation comparison between the nacre-like structure, laminated structure and monolithic structure under static or specific low-speed loads, and the sketch for a speculation that whether the nacre-like architecture is still a template for impact resistance in a wide range of impact velocities.

Bioinspired structural design attracts intense interest in achieving superior impact resistance in protective structural materials^1–12^, which enables orders-of-magnitude improvements of energy dissipations by tuning deformation modes dictated by internal architectures^13–17^. A well-known model for bioinspiration is the nacre from mollusk shells, which is generally regarded as one of the toughest body armors in nature^18–20^. The nacre is composed of hard mineral tablets and soft biopolymers in a three-dimensional “brick-and-mortar” arrangement. Studies have shown that the toughness of nacre is about 3000 times higher than that of its mineral constituents^21–25^, but it is worth noting that these studies are confined to quasi-static conditions. Under specific-speed dynamic loads, synthetic nacre-like structures duplicating the “brick-and-mortar” arrangement of nacre show superior energy-dissipation ability^10,26–28^, e.g., Yin et al.^10^ reported that such nacre-like structures can boost impact resistance by two to three times compared to traditional laminated structures and stiff monolithic structures, but these responses are observed at a specific low impact velocity of about 2.3 m s^−1^. Therefore, the current reports on the superior energy dissipation ability of nacre-like structures are often limited to a narrow range of low-impact velocities. Meanwhile, a natural phenomenon ignored by many people is that nacre was often smashed by predators with a rising striking speed (Fig. 1a), for example, the abalones can be smashed by mantis shrimps with the striking speed ranging from 14.7 to 23.5 m s^−1^ ^29,30^. The drastic difference in the impact resistance of nacre-like structures under different impact velocities triggers us to speculate, with the increase of impact velocity, whether the nacre-like architecture is still a suitable design template for impact-resistant structures, for example compared to traditional laminated structures and stiff monolithic structures, needs to be further explored.

As shown in Fig. 1b, for stiff monolithic structures, perforation failure often occurs under impact loads, which leads to limited energy dissipation due to localized deformations^26,31^. Laminated structures often fail in a brittle fashion with multiple catastrophic radial cracks and interlayer delamination under impact, but energy-absorbing capabilities of the interlayer were less exploited due to that shear deformation between adjacent plain layers is limited under lower loads^10,32,33^. For nacre-like structures, previous studies have shown that the impressive toughness enhancement originates from the tablet sliding mechanism^10,34–36^, where millions of tablets slide on one another over large volumes under tensile forces or low-velocity impact loads. Due to the energy-dissipation capacities of different structures depending on their specific failure modes, studies have reported that, upon static loads or a specific low-speed impact, the structures with energy-dissipation abilities from low to high are monolithic structures, laminated structures and nacre-like structures, respectively^10,11,26,27,37–42^. We have collected experimental data on energy dissipations versus loading velocity for nacre-like structures, traditional laminated structures and stiff monolithic structures as shown in the map of Fig. 1c. So far, the results in Fig. 1c are validated with the argument that the nacre-like structure is the winner during the energy dissipation comparison between the nacre-like structure, laminated structure and monolithic structure under static or specific low-speed loads. However, with the increase in impact velocity, the comparison of the failure modes and energy-dissipation abilities between different structures remains unclear (Fig. 1c), the nacre-like structure may not always be a template for impact-resistant structures. Besides the above single bioinspired architecture design, the hybrid design combing multiple bioinspired architectures also attracts intense interest in achieving enhanced impact resistance, for example, the hybrid laminated glass^10^, the hybrid beam^43^, hybrid gradient bioinspired materials^44^ and the hybrid gradient metal^45,46^. However, the current hybrid designs are often trial and empirical combinations of multiple structures and are confined to the cases of static loads or a specific impact velocity^10,43–46^, while there is a lack of universal hybrid design criteria for improving impact resistance at a wide range of impact velocities.

In this study, by laser engraving, drop tower testing and finite element simulations (FEM), we compare the failure modes and the energy-dissipation abilities between nacre-like structures, laminated structures and monolithic structures under different impact velocities. With the increase of impact velocity, the nacre-like structures show the best impact resistance in the initial low-velocity interval, while the laminated structures show better energy-dissipation abilities when the impact velocity exceeds a critical value. This anomalous phenomenon remains under different geometry sizes (i.e., layer thickness, tablet aspect ratio, sample thickness and layer number) and boundary conditions (i.e., contact radius and bending length), while the value of critical velocity may vary. Detailed analyses for failure modes show that the tablet sliding occurs earlier and wider in nacre-like structures at low impact velocity, while delamination and cracks in laminated structures become more significant with the increase of impact velocity. Based on the specific velocity-dependent impact resistance of different structures, we propose a universal hybrid design strategy combing the laminated architecture and the nacre-like architecture to successfully achieve optimal impact resistance in a wide range of impact velocities.

## Results

### Investigation of velocity-dependent impact performances

**Fig. 2:**
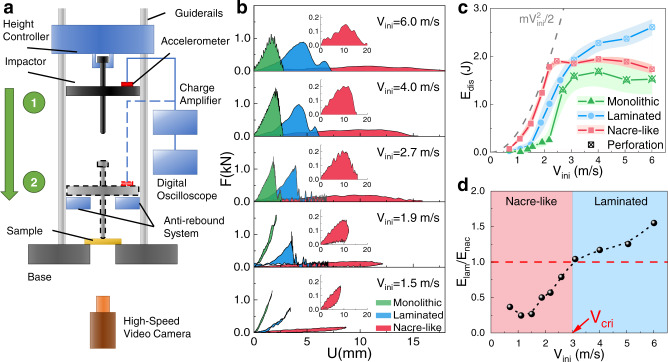
Impact resistance of the nacre-like structure under different impact velocities. **a** Schematic of the drop-tower test system equipped with a high-speed camera. **b** The force *F*-displacement *U* curves of the monolithic samples, laminated samples, and nacre-like samples under different impact velocities *V*_*ini*_. The insets show magnified views for force-displacement curves of the nacre-like samples. **c** The total energy dissipation *E*_*dis*_ - *V*_*ini*_ curves in different hierarchical structures. The crossed symbols represent the perforation of the specimen. The colored shadows are error bars representing the standard deviations of at least three replicate measurements. **d** The ratio of energy dissipation of laminated structure *E*_*lam*_ to that of nacre-like structure *E*_*nac*_ under different *V*_*ini*_.

To investigate the effects of impact velocity on the impact performances, we performed drop tower tests on the nacre-like structures, laminated structures and monolithic structures under different impact velocities. The specimens ($60\times 60\times 1.4$ mm^3^) with the nacre-like structure were made of five engraved 200-μm-thick borosilicate glass sheets and four 100-μm-thick polymeric interlayers (Ethylene-vinyl acetate) in a three-dimensional staggered “brick-and-mortar” arrangement akin to natural nacre (Supplementary Fig. 1). The borosilicate glass sheets were engraved into 1600 Voronoi polygonal tablets by laser to mimic the contours of tablets in nacre from abalone shell^35^. Further, the engraved glass sheets were carefully laminated with polymeric interlayers to form a three-dimensional staggered brick-and-mortar arrangement similar to natural nacre, where the overlap areas between tablets in adjacent layers cover about 1/3 of the tablet area. It is worth mentioning that the reason why we choose the nacre-like glass as the test object is that the mechanical properties of its tablets and interfaces are similar to that of natural nacre^35^, where the glass tablets are brittle and the polymeric interlayers are highly deformable. Due to the precise control over the size and arrangement of tablets, as well as the high deformability, strain hardening, strong adhesion and high energy absorption of the polymeric interlayers, the nacre-like samples are capable of achieving tablets sliding over large areas to enhance impact resistance. In addition, compared to other bioinspired composites with nacre-like structures, our synthetic nacre-like glass samples fulfill the requirements for this work, including the precise control of internal size and architecture, the reproduction of large-scale tablet sliding and the mass production of bulk samples with designed structures. For specimens with laminated structures, five intact 200-μm-thick borosilicate glass sheets were alternately laminated with four ~100-μm-thick polymeric interlayers. The monolithic borosilicate glass sheet of the same size as nacre-like and laminated specimens was used as a monolithic structure. The drop tower test machine equipped with a high-speed video camera was used to perform impact tests on simply supported specimens with different architectures (Fig. 2a). The details of the fabrication and experimental setup are provided in “Method” section and Supplementary Discussion 1.

The force-displacement curves in Fig. 2b show that at different initial impact velocities, the monolithic structures and laminated structures have high strength but low deformations and both fail in a brittle fashion when penetrated. With the increase of initial impact velocity, out-of-plane deformations of laminated structures increase, while the monolithic structures retain minimal failure displacement. Meanwhile, the force-displacement curve of the laminated structures oscillates violently after descending from the peak, which is due to the delamination and multiple cracks formation. By contrast, the nacre-like structures exhibit more ductile responses with large deformations and low strength. With the initial impact velocity increasing, the out-of-plane deformation increases and the contact force first increases and then decreases. Moreover, the step-like fluctuations in curves reveal the existence of large-scale tablet sliding and localized microcracks formation in nacre-like structures. It is worth noting that at the impact velocity of 2.7 m s^−1^, the monolithic structures and the nacre-like structures are penetrated but the laminated structure is still intact, which is due to the polymeric interlayers holding the fragments and maintaining the integrity of the laminated structures. This phenomenon indicates that at a relatively low impact velocity, the deformation and energy dissipation capabilities of the laminated structures were less exploited, while the nacre-like structure performed to the saturation of its energy-dissipation capabilities at a relatively high impact velocity.

Further, the colored areas in Fig. 2b represent the energy dissipations *E*_*dis*_ by the irreversible deformations of specimen at the end of an impact event. Figure 2c shows the energy dissipation *E*_*dis*_—impact velocity *V*_*ini*_ curves for the nacre-like structures, laminated structures and monolithic structures, respectively. Results show that compared to the nacre-like structures and the laminated structures, the monolithic structures have the lowest *E*_*dis*_ at given different initial impact velocities. For the laminated structures, *E*_*dis*_ increases slowly and then rapidly with the increase of *V*_*ini*_, and it maintains moderate increases even after *V*_*ini*_ exceeds the critical penetration velocity. By contrast, nacre-like structures show highest *E*_*dis*_ compared with the laminated structures and the monolithic structures within a narrow range of impact velocity less than 3 m s^−1^, while the laminated structures exhibit higher impact resistance than the nacre-like structures when impact velocity exceeds 3 m s^−1^. Further, Fig. 2d shows the *ratio E*_*lam*_*/E*_*nac*_ between the energy dissipation of laminated structures and that of nacre-like structures under different *V*_*ini*_. Results show that the *E*_*lam*_*/E*_*nac*_ significantly increases with increasing initial impact velocity and is greater than 1 when the impact velocity exceeds the critical value *V*_*cri*_ of 3 m s^−1^. Counter-intuitive as it may seem, our experiments found that nacre-like structures show superior energy-dissipation ability only in a narrow range of low-impact velocities, while they exhibit lower impact resistance than laminated structures when impact velocity exceeds a critical value.

### Characterization of velocity-dependent failure modes

**Fig. 3:**
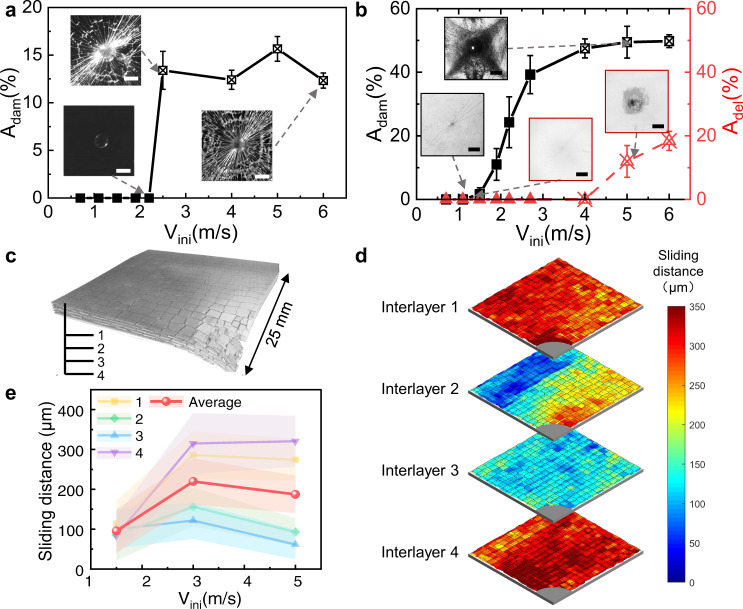
Energy dissipation modes for nacre-like structures and other structures under different *V*_*ini*_. **a** The proportion *A*_*dam*_ of fracture area for the monolithic sample under different *V*_*ini*_. The insets show the damage patterns of the impacted samples. Scale bar: 10 mm. **b** The *A*_*dam*_ in hard layers and the proportion *A*_*del*_ of delamination area in soft phase for the laminated samples under different *V*_*ini*_. The insets show damage morphologies under *V*_*ini*_. Scale bar: 10 mm. **c** Micro-CT scan 3D reconstruction of a quarter of the nacre-like sample. **d** Maps of the sliding distance for each interlayer in the nacre-like panel under *V*_*ini*_ = 3 m s^−1^. **e** The sliding distances - *V*_*ini*_ curves for each interlayer in the nacre-like samples. All the error bars represent the standard deviations of at least three replicate measurements.

We further characterized the failure modes that occurred during above impact experiments to gain a better understanding of the energy dissipation mechanisms for the nacre-like structures, laminated structures and monolithic structures under different impact velocities. The nondestructive evaluations including photography, X-ray radiography and micro-CT were used to assess the failure situations (The detail can be found in Supplementary Discussion 2). As shown in Fig. 3a and Supplementary Figs. 2, 3, the main failure mode of the monolithic structures is the catastrophic and explosive failure of the bulk glass^47^, where multiple radial cracks emanate from the impact point to the edges of the panel and the circumferential cracks occur due to bending deformations. Further, for the monolithic structure, the proportion of failure area *A*_*dam*_ in the monolithic glass remains zero before cracks occur and then increases with the initial impact velocity *V*_*ini*_, and the *A*_*dam*_ increases to 13.4% and remains constant after the monolithic structures are penetrated (Fig. 3a). For the laminated structures, the failure modes are intralayer explosive failures and interlayer delamination, where long radial and circumferential cracks occur in plain glass layers and enlarged delamination area exists in the polymeric interlayers underneath the impactor (Fig. 3b and Supplementary Fig. 4). Meanwhile, Fig. 3b shows the percentages of the intralayer damage area *A*_*dam*_ and the interlayer delamination area *A*_*del*_ in the laminated structures under different *V*_*ini*_. The *A*_*dam*_ and *A*_*del*_ in the laminated structures remain zero before the cracks and delamination occur and then increase with the initial impact velocity. When *V*_*ini*_ exceeds the value of 1.5 m s^−1^, the failure modes in intralayer glass sheets change from the radial cracks to the radial and circumferential cracks, and the *A*_*dam*_ increases to about 50% as the *V*_*ini*_ increases. Moreover, the interlayer delamination occurs when *V*_*ini*_ exceeds 4 m s^−1^, and the *A*_*del*_ increases to 18.4% as the *V*_*ini*_ increases. These impact velocity-dependent trends of failure modes are similar to that of energy dissipations in laminated structures (Fig. 2c), where the multiple long radial and circumferential cracks and the enlarged delamination area give full play to the energy dissipation abilities of the laminated structures under high impact velocity. For the nacre-like structures, the main failure mode is the tablet sliding, where a large number of tablets can slide on one another over large volumes, along with large-scale nonlinear shear deformations in interfaces (Fig. 3 and Supplementary Fig. 5). Here, three-dimensional microtomography perspectives for a quarter of the impacted samples were reconstructed by micro-computed tomography (micro-CT) (Fig. 3c), which provides a comprehensive map of the micromechanics of deformation in the nacre-like structures under different impact velocities. Further, Fig. 3d and Supplementary Fig. 6 show the maps of sliding distances of each interlayer in the nacre-like samples under different impact velocities. The sliding distances in each interlayer of the nacre-like structures under *V*_*ini*_ = 3 m s^−1^ were much larger and more homogenously distributed, while the distributions of larger sliding distances become saturated near the impactor when *V*_*ini*_ = 5 m s^−1^. Based on the distributions of sliding distances, we calculated the average values of sliding distances in each interlayer and the average sliding distances in nacre-like structures (Fig. 3e). For each interlayer in the nacre-like structures, the average values of sliding distances increase first and then tend to be constant when the *V*_*ini*_ exceeds 3 m s^−1^. These trends indicate that the nacre-like structure can fully activate the wider tablets sliding in a narrow range of low *V*_*ini*_, but the tablets sliding becomes saturated or cannot be further developed when the *V*_*ini*_ exceeds a critical value. These impact velocity-dependent trends of tablet sliding are in good agreement with that of energy dissipations in nacre-like structures (Fig. 2c). Therefore, the tablet sliding in nacre-like structures occurs earlier and wider at low impact velocities and thus the nacre-like structures show superior energy-dissipation ability, while at excessive impact velocities the energy dissipations of delamination and cracks in laminated structures become higher than that of saturated tablets-sliding in nacre-like structures.

### Analysis of velocity-dependent energy-dissipation mechanisms

**Fig. 4:**
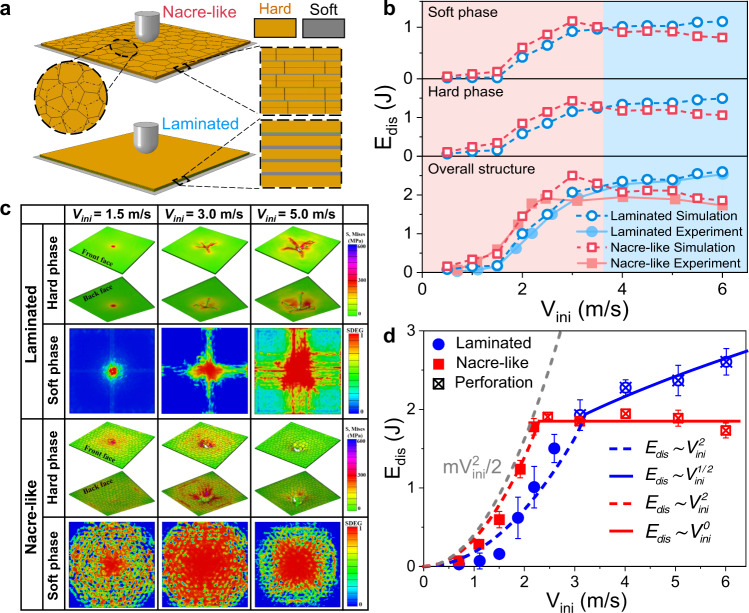
Analysis of the critical impact velocity. **a** Finite element model for the nacre-like and laminated structures under impact loading. **b** The *E*_*dis*_–*V*_*ini*_ curves for the overall nacre-like structures, the overall laminated structures, the hard phases and the soft phases in the nacre-like and laminated structures. **c** The deformation maps for the soft layers (scalar stiffness degradation variable (SDEG) field) and the hard layers (von Mises stress field) in the nacre-like and laminated structures under different *V*_*ini*_, where SDEG = 0 represents zero failure while SDEG = 1 stands for the complete failure. **d** Scaling laws between the energy dissipation *E*_*dis*_ and the impact velocity *V*_*ini*_ for the laminated and the nacre-like structures.

To explicate the underlying mechanisms occurring in the nacre-like and laminated structures under different *V*_*ini*_, we simulate the impact performances using nonlinear finite element method. Figure 4a shows the finite element models for the nacre-like and laminated structures under impact loads, and the details can be seen in “Method” section and Supplementary Discussion 4 (Supplementary Fig. 7). Failure morphologies predicted via simulations are in good agreement with experimental results (Supplementary Fig. 8a), where lots of tablets slide on one another over large volume in nacre-like structures and the radial cracks and delamination occur in laminated structures. Meanwhile, good agreement between force-displacement curves (Supplementary Fig. 8b) obtained from experiments and simulations implies that our finite element model can well reveal the dynamics of energy dissipation in the laminated and nacre-like design under different impact velocities. In addition, simulated energy dissipation *E*_*dis*_–impact velocity *V*_*ini*_ curves in Fig. 4b demonstrate that nacre-like structures show superior energy-dissipation ability in the narrow range of low impact velocities, while they exhibit lower impact resistance than that of laminated structures when impact velocity exceeds a critical value of about 3 m s^−1^, which is consistent with the experimental value in Fig. 2c. Further, to explicate the internal mechanisms of above energy-dissipation trends and the critical impact velocity, Fig. 4b shows the energy dissipations of the soft EVA interlayers and the hard glass layers in nacre-like structures and laminated structures under different *V*_*ini*_. Similar to the trend of total energy dissipation changing with *V*_*ini*_, the *E*_*dis*_ of soft EVA layers and hard glass layers in nacre-like structures is higher than that in laminated structures at low *V*_*ini*_, whereas the *E*_*dis*_ of soft EVA layers and hard glass layers in laminated structures become larger than that in nacre-like structures when the *V*_*ini*_ exceeds a similar critical value of about 3 m s^−1^. Thus, the critical impact velocity originates from the differences between velocity-dependent energy dissipation abilities of hard phases and soft phases in nacre-like structures and laminated structures. Furthermore, Fig. 4c shows the failure patterns in the hard glass layers and the soft EVA layers of the nacre-like structures and laminated structures under different *V*_*ini*_. At a low *V*_*ini*_, the soft interlayers of nacre-like structures show larger and more homogenously distributed interfacial sliding patterns, which are consistent with previous findings that tablet sliding is the main mechanism for high energy dissipation in nacre-like structures^10^. By contrast, for the laminated structures under low *V*_*ini*_, the small delamination area in the soft interlayers and the limited failure area in hard glass layers are concentrated underneath the impactor, indicating low-impact energy dissipation. However, when the *V*_*ini*_ exceeds the critical value, the tablet sliding in the nacre-like structure is saturated, while the interfacial delamination area in the laminated structure significantly enlarges, indicating enhanced interlayer energy dissipation. In addition, for the nacre-like structures under a high *V*_*ini*_, the high-stress area appears underneath the impactor and the perforation prefers to occur in the glass layers, but the laminated design distributes the stress over a larger volume of structure and avoids stress localization. Meanwhile, as shown in Supplementary Fig. 9, with the impact velocity *V*_*ini*_ increases, the proportion of tablet-sliding induced failure area (*A*_*SF*_*/A*_*0*_) in each interlayer of nacre-like structures increases first and then slightly decreases as the *V*_*ini*_ exceeds a critical value, which is in good agreement with the trend of energy dissipation in Fig. 4b. Hence, the distinct velocity-dependent failure modes in soft interlayers and hard layers of the nacre-like structures and laminated structures result in the critical impact velocity.

Further, we built the scaling laws between the energy dissipation and impact velocity for the laminated structure and the nacre-like structure based on experimental results (Fig. 4d), while it is difficult to perform fully analytical investigation of critical velocity due to the complexity of the failure process in laminated and nacre-like structure during impaction. In the low impact-velocity range where the structures are not perforated, the fitted scaling laws for the energy dissipation of the laminated and nacre-like structures under different impact velocities are ${E}_{dis}\sim {V}_{ini}^{2}$, which is proportional to the initial kinetic energy $m{V}_{ini}^{2}/2$ (see gray line in Fig. 4d). In the speed range before the perforation occurs, the energy-dissipation modes of tablet sliding in nacre-like structure are more easily to occur than that of the delamination and crack propagation in laminated structure, thus the nacre-like structure can completely dissipate the initial kinetic energy. Further, when the *V*_*ini*_ exceeds a critical value, the nacre-like structure remains nearly constant energy dissipations under different impact velocities (${E}_{dis}\sim {V}_{ini}^{0}$) due to that the discontinuous glass layers result in the localized perforation and the tablet sliding is saturated. For the laminated structure, the scaling law between the energy dissipation and the impact velocity satisfies ${E}_{dis}\sim {V}_{ini}^{1/2}$, which can be explained by the crack patterns dependent on impact velocity^47,48^. In the range of the *V*_*ini*_ that exceeds the critical value, the perforation occurs and the main energy-dissipation modes in laminated structures include delamination and radial crack propagation, and the *E*_*dis*_-*V*_*ini*_ curves of interlayer delamination show similar trends to that of the radial crack propagation in glass layers based on our simulations (Fig. 4b). In Griffith’s theory of brittle fracture, the energy dissipated by radial cracks in glass layers is ${E}_{dis}=2n\varGamma h{r}_{f}$, where $\varGamma$is the material fracture (surface) energy, *h* is the thickness, ${r}_{f}$ is the length of crack propagation and *n* is the number of radial cracks. Because the impact velocity considered in this work is much smaller than the wave speed, the time scale is much longer with respect to the propagation time of the strain in the medium, thus the cracks can propagate to a steady constant ${r}_{f}$, and the number of radial cracks satisfies a scaling law of $n\sim {V}_{ini}^{1/2}$ under different *V*_*ini*_^47,48^. Therefore, in the speed range above a critical value, the energy dissipation for the laminated structure satisfies a scaling law of ${E}_{dis}\sim {V}_{ini}^{1/2}$. Based on above fitted scaling laws between the energy dissipation and impact velocity for the laminated structure and the nacre-like structure, we found that there is always a critical velocity above which the nacre-like structures exhibit lower impact resistance than that of laminated structures.

### Universality of the phenomenon existing a critical impact velocity

**Fig. 5:**
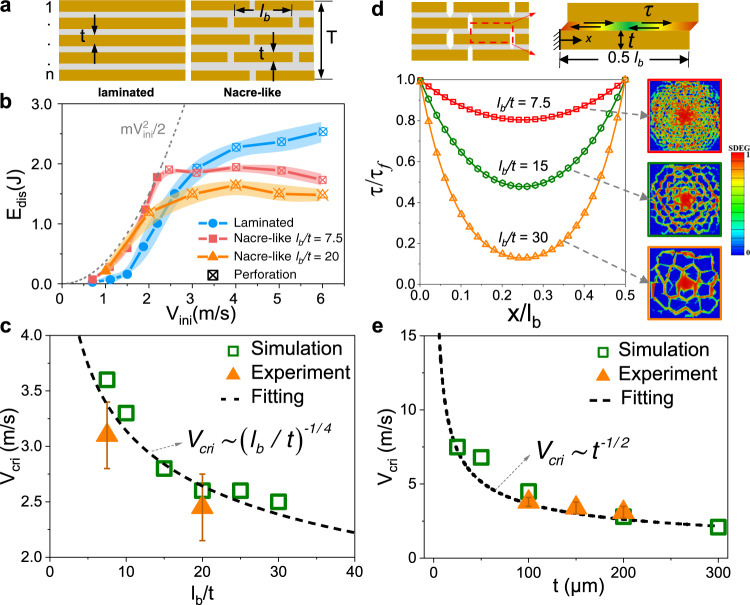
Effects of the structural geometry on the critical impact velocity. **a** Schematic of key geometry parameters in laminated and nacre-like structures. **b** Experimental *E*_*dis*_- *V*_*ini*_ curves for the laminated structures and the nacre-like structures with different tablet aspect ratios *l*_*b*_*/t*. **c** The critical impact velocity *V*_*cri*_ as function of *l*_*b*_*/t*. **d** Analysis for shear stress distribution $\tau$ in the nacre-like structures with different *l*_*b*_*/t* by the shear-lag model. The insets show the simulated deformation maps of the soft phase (scalar stiffness degradation variable (SDEG) field) in the nacre-like structures with different *l*_*b*_*/t* under *V*_*ini*_ = 1.5 m s^−1^. **e** The *V*_*cri*_ as functions of the layer thickness *t*. All the error bars represent the standard deviations of at least three replicate measurements.

To further prove the universal existence of the anomalous inapplicability of nacre-like architecture as an impact-resistant template in a wide range of impact velocities, we performed more comprehensive investigations on impact performances of the laminated and nacre-like structures with different structural sizes and boundary conditions (Fig. 5a and Supplementary Discussion 5–7). Based on our previous findings that the aspect ratio of tablets is key to the tablet sliding mechanism in nacre-like structures^17,36^, we performed experiments, simulations and theoretical analyses to reveal the effects of the aspect ratio of tablets *l*_*b*_/*t* (*t* = 200 μm) on the critical impact velocity *V*_*cri*_ (Fig. 5b–d). Experimental results show that with the increase of *l*_*b*_/*t*, the energy dissipation of nacre-like structure decreases (Fig. 5b and Supplementary Fig. 10), which results in the decrease of *V*_*cri*_. Further, results in Fig. 5c show that the *V*_*cri*_ significantly increases with the decrease of *l*_*b*_/*t* and satisfies a fitted scaling law of ${V}_{cri}\sim {({l}_{b}/t)}^{-1/4}$. This trend originates from the fact that as *l*_*b*_/*t* increases, the deformations of glass layers become concentrated underneath the impactor, the interlayer sliding is localized at the edges of tablets and the average *A*_*SF*_*/A*_*0*_ in each interlayer decreases (Supplementary Fig. 11). Theoretical analysis by the nonlinear shear-lag model^36^ shows that the distribution of interfacial shear stress becomes highly localized with *l*_*b*_/*t* increases (Fig. 5d and Supplementary Discussion 5), which leads to that the interlayer sliding localized at the edges of tablets, the energy dissipation decreases and thus the *V*_*cri*_ decreases. Further, results in Fig. 5e and Supplementary Discussion 5 show that the *V*_*cri*_ increases significantly with decreasing layer thickness *t* and satisfies a fitted scaling law of ${V}_{cri}\sim {t}^{-1/2}$. This also indicates that the critical velocity can become much larger when the characteristic size of the nacre-like structure decreases to nanoscale^18,35^, which may explain why the natural nacre with nanoscale multi-layered structures could not keep superior impact resistance when the impact velocity is above ranging from 14.7 to 23.5 m s^−1^ ^29,30^. In addition, experimental and simulated results show that there is always a critical velocity above which the nacre-like architecture is no longer a suitable design template for impact-resistant structures, in a wide range of sample thickness, layer numbers, contact radius and bending length (see details in Supplementary Discussion 6, 7 and Supplementary Figs. 12–19). Further, drop tower testing for 3D printed samples with designed nacre-like and laminated structures also show that nacre-like structures show superior energy-dissipation ability only in a narrow range of low impact velocities, while they exhibit lower impact resistance than that of laminated structures when impact velocity exceeds a critical value (see details in Supplementary Discussion 8 and Supplementary Fig. 20). It is worth mentioning that in this work we mainly focus on the energy dissipations of the bending or tension-induced deformations over large volumes (i.e., crack propagation, delamination and tablet sliding) and ignore the limited energy-dissipations induced by deformations localized at contact point. These bending or tension-induced energy-dissipation modes are biologically relevant, where the designed nacre-like structures duplicate the three-dimensional “brick-and-mortar” arrangement of natural nacre and reproduce the bending-induced tablet sliding mechanism in natural nacre^10,20,35,36^. Since the critical impact velocity is rooted by different failure modes leading to different impact velocity-dependent energy-dissipation capacities, we believe that it may not be an occasional occurrence but universal and can be expanded to general bioinspired layered structures.

### Design of impact-resistant hybrid structures

**Fig. 6:**
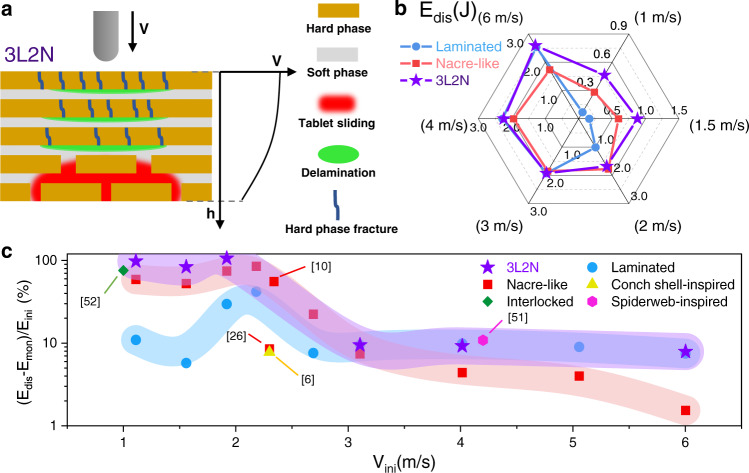
Hybrid architectural panel design. **a** Schematic of the hybrid architecture’s design considering the impact velocity distribution along the cross-section of panel, where 3L2N represents three layers of the laminated structure and two layers of the nacre-like structure. **b** Energy dissipation *E*_*dis*_ of the laminated samples, the nacre-like samples and the 3L2N samples under different impact velocities. **c** Impact energy dissipation comparison between the designed bioinspired architectures in this work and reported bioinspired architectures under a wider range of impact velocities, where *E*_*mon*_ represents the energy-dissipation of monolithic structure and *E*_*ini*_ represents initial impact energy. All the data points represent the average values of at least three replicate measurements.

The fact that the nacre-like structure shows higher energy dissipation below a critical impact velocity while lower energy dissipation above it than that of the laminated structure indicates that we could achieve a hybrid design strategy combing the advantages of different structures to enhance impact resistance in a wide range of impact velocities. Considering that the impact velocity attenuates along the loading direction of a panel^49,50^, we design a hybrid panel composed of the laminated structures in front high velocity region and the nacre-like structures in back low velocity region (Fig. 6a and Supplementary Fig. 21). This hybrid design can achieve multiple failure modes including the long radial and circumferential cracks and enlarged delamination in laminated structures as well as the tablets sliding in nacre-like structures (Fig. 6a). We design six hybrid configurations combining different layers of nacre-like structures and laminated structures to investigate the impact resistance under different impact velocities (Supplementary Fig. 21a). The radar chart is used to give an intuitive visualization for the impact resistance of the hybrid designs under different impact velocities (Fig. 6b and Supplementary Fig. 21b). Results show that the hybrid design with the three laminated layers as the front layers and the two nacre-like layers as the back layers (3L2N) show optimal impact resistance in a wide range of impact velocities, where the 3L2N design not only has similar impact resistance to the laminated structure at high velocity interval, but also achieves higher energy dissipation than the nacre-like structures at low velocity interval. Further, Fig. 6c shows that under a wider range of impact velocities, the impact resistance of the designed 3L2N structure is superior to that of simple nacre-like structure, laminated structure and other reported architectures, such as conch shell-inspired structure, interlocked structure and spiderweb-inspired structure^6,10,26,51,52^. Therefore, based on the specific velocity-dependent impact resistance of different structures, we believe this hybrid design strategy combing the laminated architectures and the nacre-like architectures can successfully achieve optimal impact resistance in a wide range of impact velocities.

## Discussions

In summary, by an integrated approach combing laser engraving, drop tower testing and simulation, we found that the superior impact resistance of the nacre-like structure only manifests itself in a narrow range of low-impact velocities, while the laminated structure is a better impact-resistant template than the nacre-like structure when impact velocity exceeds a critical value. This new phenomenon originates from that the tablets sliding are fully activated and saturated over large areas of nacre-like structure at a narrow range of low-impact velocities, while the energy dissipation of the delamination and cracks in laminated structures become more significant at high-impact velocity interval. Under different structural sizes, boundary conditions and material compositions, the finding of the anomalous inapplicability of nacre-like architecture as an impact-resistant template in a wide range of impact velocities remains valid, while the value of critical velocity may vary. Based on our built mechanical models and scaling laws, we revealed that the critical velocity can become much larger when the characteristic size of the nacre-like structure decreases to nanoscale of natural nacre^18,35^, which may explain why the natural nacre with nanoscale multi-layered structures couldn’t keep superior impact resistance when the impact velocity is above ranging from 14.7 to 23.5 m s^−1^^29,30^. This also inspired us that in order to reproduce the superior impact resistance of natural nacre, it is necessary to ensure that the internal microstructure size of bulk artificial nacre is the same order as that in natural nacre^20^. Further, we propose a hybrid structure design strategy in which each structure is placed to have the highest energy dissipation as the impact velocity decays along the loading direction. Such strategy can combine the advantages of nacre-like and laminated structures at different impact velocities to achieve optimal impact resistance over a wide range of impact velocities. Although this study examined the effects of impact velocity on the energy-dissipation abilities of nacre-like structures, laminated structures and monolithic structures specifically, the proposed universal hybrid structure design strategy combing the velocity-dependent advantages of various structures can be tailored and adapted to enhance impact resistance of future protective structural materials in a wide range of impact velocities.

## Methods

### Preparation of multilayered glass panels

Nacre-like composite comprises five borosilicate glass (Gous Optics Co., Ltd., China) as hard phase and four Ethylene-vinyl acetate copolymer films (Caida Co., Ltd., China) as the adhesive matrix. Each borosilicate glass sheet is first engraved and separated into Voronoi polygonal tablets by laser-induced dicing system (Delphi Laser Co., Ltd., China). Then the glass sheets were carefully aligned and laminated with Ethylene-vinyl acetate interlayers. The ethylene-vinyl acetate copolymer we used is a thermoplastic material with low strength and high shear strain at failure, hot pressing it can melt to form excellent adhesion to the glass. The laminated plates were fabricated with the same procedure without laser engraving. Full details of the composite parameters and preparation process are shown in Supplementary Discussion 1–3.

### Droptower impact tests

The impact resistance characteristics for samples under different impact velocities were investigated using a droptower impact system (MTS Industrial Systems) (Fig. 2a). The specimens were simply supported at their periphery on a 5 mm wide shoulder machined in the frame. A hemispherical impactor with a diameter of 10 mm and 0.75 kg in mass is dropped from different heights ${h}_{0}$ to obtain the impact velocity of 0.7–6.0 m s^−1^. The initial impact velocity ${V}_{{{{{{\rm{ini}}}}}}}$ is calculated from ${V}_{{{{{{\rm{ini}}}}}}}=\sqrt{2g{h}_{0}}$, here *g* is the standard gravity. The acceleration data $a(t)$ of the impactor during impact is collected by a piezoelectric acceleration sensor. The displacement *U* is calculated through the equation $U(t)={\int }_{0}^{t}V(\tau )d\tau$, where $V(t)={V}_{{{{{{\rm{ini}}}}}}}+{\int }_{0}^{t}a(\tau )d\tau$, and the contact force *F* of the impactor is calculated from $F=ma$. The energy dissipation *E*_*dis*_ is the area included inside the loop of the force-displacement curves. The destruction of the sample during the impact test was filmed from the back of the impact face with a high-speed camera (5KF20, Revealer, China). The acquisition rate was 5000 frames s−^1^.

### Characterization of failure modes

The delamination characteristics of laminated structures were analyzed by X-ray radiography using an X-ray inspection system (Dage Quadra7, Nordson, UK). Images taken are inverted to black and white to highlight the delaminated area. Delamination was quantified by counting the proportion of dark areas in the total image. The nacre-like structure damage of table-sliding was analyzed by Micro-CT (Xradia 520 Versa, Zeiss, Germany). The three-dimensional model was rendered with 3D visualization software (ORS, Dragonfly, Canada). The changing distance between the center points of the tablets in the adjacent layers after the impact of the sample was used as the sliding distance in the brick overlap area. Due to the central symmetry of the impacted sample, a quarter of the sample was scanned to represent the entire sample (see details in Supplementary Discussion 2).

### Finite element modeling

To analyze the impact responses of nacre-like structures and laminated structures under different impact velocities, we developed 3D nonlinear finite element models using the commercial software ABAQUS v6.14. The nacre-like structure comprises random staggered arrangements of stiff glass tablets bonded by thin layer of EVA, and the glass layer made of random polygonal tablets mimicking natural nacre was generated through Voronoi technology. The laminated structure contains complete glass panels bonded by EVA layers. The stiff glass with the failure strength of 150 MPa, isotropic bulk modulus of 75 GPa, Poison ratio of 0.2 and density of 2.23 g cm^−3^ were simulated using brittle cracking model in ABAQUS. The soft EVA layers with the failure strength of 1.75 MPa and critical energy release rate of 2 N mm^−1^ were modeled by cohesive elements with a trapezoidal cohesive law, which represents ideal elastic-plastic nonlinear behaviors due to tablet sliding and EVA elongation. In the simulation, the nacre-like structure and laminated structure are simply supported by a rigid frame, and a cylindrical impactor with 0.75 kg in mass is treated as a rigid body and is fully constrained except in the loading direction. The material and geometric parameters in the Abaqus model are consistent with experimental setups, and details can be seen in Supplementary Discussion 3, 4.

## Supplementary information

Supplementary Information

## Data Availability

The data generated in this study are provided in the Supplementary Information Files.
