# Supplementary material for: Anomalous inapplicability of nacre-like architectures as impact-resistant templates in a wide range of impact velocities
Source: Nat Commun. 2022 Dec 13;13:7719. doi: 10.1038/s41467-022-35439-3 (PMC9747917; doi:10.1038/s41467-022-35439-3)
Supplement: Supplementary file 1 — Supplementary Information [file 41467_2022_35439_MOESM1_ESM.pdf]

Supplementary Information

Anomalous inapplicability of nacre-like architecture as an impact-resistant template in a wide range of impact velocities

Zhang et al.

This PDF file includes:

Supplementary Discussion 1: Fabrication of multilayered glass panels.

Supplementary Discussion 2: Nondestructive evaluation of the panels' damage.

Supplementary Discussion 3: Mechanical properties of components

Supplementary Discussion 4: Finite element modeling of impact responses.

Supplementary Discussion 5: Effects of the aspect ratio of tablets and the layer thickness.

Supplementary Discussion 6: Effects of the sample thickness and the layer number.

Supplementary Discussion 7: Effects of the contact radius and the bending length.

Supplementary Discussion 8: Drop tower testing for 3D printed samples.

Supplementary table 1

Supplementary Figure 1 to Figure 21

Supplementary References

Supplementary Discussion 1: Fabrication of multilayered glass panels

The multi-layered glass panels were composed of hard phases made of borosilicate glass (Gous Optics Co., Ltd., China) and soft interface made of EVA thermoplastic film (Caida Co., Ltd., China) (Supplementary Fig. 1a). The hard phases of the nacre-like panels were made with Voronoi polygon glass platelets to mimic the nacre's tablet structure. Voronoi diagram was a well-known computational geometry comprised of seeds, where every seed inside the polygon consisting of all points of the plane was closest to that seed. We generated random Voronoi diagram from the grid formation (with side length *L*) by allowing the seeds to move within the blue circle (with radius *R*) randomly (Supplementary Fig. 1b). The original grid formations of adjacent layers' Voronoi diagram were offset $0.18\times L$ in the X and Y directions, for the overlap of nacre's tablets from adjacent columns covers about 1/3 of the tablet surface area. The borosilicate glasses were engraved Voronoi polygon design pattern using a laser-induced dicing system (Delphi Laser Co., Ltd., China). Then the engraved glasses were glued with polyimide tape and gently bent to form Voronoi platelets along with the laser-engraved weak interfaces.

The bent glass plates were first laminated with EVA thermoplastic film to form two-layer plates. The two-layer pre-laminates were constrained by a rigid frame and assembled in a vacuum oven under 18 kPa pressure at 130 °C for 1.5 hours. Then the polyimide tape was removed from the two-layer laminates. The assembly was laminated with the same procedure again to fabricate five-layered laminates. The Voronoi platelets of panel's each layer can be observed under a microscope by focusing on different layers (Supplementary Fig. 1c). The overlap of platelets between adjacent layers was shown in the microscopic image of the plate's cross-section (Supplementary Fig. 1d). The laminated plates were fabricated with the same procedure without laser engraving (Supplementary Fig. 1a).

Supplementary Discussion 2: Nondestructive evaluation of the panels' damage

The nondestructive evaluation includes photography, X-ray radiography and micro-CT. Laminated structure damage mainly includes fragmentation damage to the hard phase and delamination damage to the soft phase. Fracture damage to hard phases characterized by photography. All photographs were taken under the same lighting conditions and binarized at the same parameters. Supplementary Fig. 2 shows damage characterization of the monolithic samples subjected to impact at different velocities. As shown in Supplementary Fig. 3a, the impact loads result in bending deformations in the glass plate

held on a square frame, and the pattern of cracks corresponds to the global energy (i.e., elastic plus fracture) minimum[1, 2]. The coexistence of radial and circumferential cracks is observed under different impact velocities and the radius r_f of the circumferential cracks has been observed. Results in Supplementary Fig. 3b show that the r_f decreases with the increasing of impact velocity V_{ini} and satisfies a fitted scaling law of $r_f \sim V_{ini}^{-1/2}$, which is in good agreement with the results in previous work[1, 2].

Delamination damage to the soft phase is characterized by X-ray inspection system (Quadra7, Nordson Dage, UK). The X-ray was performed using a 4.5 W power at 80 kV of potential. The cracks of the impacted sample were too thin to be distinguished by X-ray radiography, but delamination can be displayed for their low density relatively due to the presence of air. Supplementary Fig. 4 shows the hard phase damage and interlayer delamination in laminated panels after impact with different impact velocities.

Micro-CT was used to analyze the damage of table-sliding through a 3D X-ray microscope (Xradia 520 Versa, Zeiss, Germany). The scans were carried out with a 60 kV accelerating voltage and an 82 mA power, resulting in a voxel size of 13 μm/pix. The high aspect ratio tomography was used to improve the scanning effect. The three-dimensional model was rendered with 3D visualization software (Dragonfly, ORS, Canada). Each platelet of samples was extracted from different datasets by applying thresholding operations (Supplementary Fig. 5). The parameters of every platelet such as volume, surface area, and center of mass can be measured automatically after the dataset was segmented. Due to the central symmetry of the impacted sample, a quarter of the sample was scanned to represent the entire sample (Supplementary Fig. 5a). The sliding distance within each interlayer was shown in Supplementary Fig. 6.

Supplementary Discussion 3: Mechanical properties of components

The mechanical properties of the glass were measured by three-point bending tests followed ASTM D790-03. The glass bending specimen is characterized by a gauge dimension of 50 mm, a width of 12.5 mm, a thickness of 0.2 mm. The samples mounted on a Material Test System were performed with a support span of 25 mm at a loading rate of 0.05 mm/min. Force-displacement curves obtained from the tests were converted to stress-strain curves, as shown in Supplementary Fig. 7a.

The shear performance of the interface between EVA and glass was measured by shear lap tests

followed ASTM C961 to simulate the impact responses of nacre-like structure and laminated structure. We conducted single lap shear tests by depositing 50-μm-thick layer of EVA over a 25 mm× 2 mm overlap area between pairs of 2 mm thick borosilicate glass substrates. The shear lap tests were carried out on a Material tension Test System to induce a simple shear state in EVA at a loading rate of 0.5 mm/min. Force-displacement curves obtained from the tests were converted to shear stress-strain curves using the surface area and thickness of the EVA layer (Supplementary Fig. 7b). The shear stress-strain curve shows that EVA has very high deformability in shear and low strength. EVA combines elastomeric response with large inelastic deformations, which is particularly well suited for nacre-like interfaces.

Supplementary Discussion 4: Finite element modeling of impact responses

For the finite element model of nacre-like structure, the Voronoi technique was employed to generate the finite element model of polygonal tablets arrangement mimicking the random distribution of mineral tablets in natural nacres. Firstly, a set number of nuclei are generated in a square area based on the principle that the distance between any two points is not less than a given distance. These nuclei are copied to their surrounding neighboring areas through translation to construct the Delaunay triangulation and the Voronoi diagram. The polygon cell in the Voronoi diagram contains all points that are closest to its nucleus than any other nuclei. Finally, the polygons outside of the square area are discarded to achieve the finite periodic Voronoi structure. The monolayer nacre-like tablets specimen in our simulation is constructed in a region of 60 mm × 60 mm, and its thickness and the number of nuclei can be designed. The nacre-like tablets FEM model is meshed by using ABAQUS solid elements (C3D8R and C3D6R) with the size of about 0.1 mm. There are two layers of element mesh in the one glass layer to capture the bending effects of plate. The zero-thickness cracks are introduced between two volumetric elements along the boundaries of the polygon tablets. The nacre-like structure is made up of five layers of stiff platelets. The adjacent plies are bonded together by the soft EVA layer. The soft EVA layers are meshed using ABAQUS solid elements (COH3D8). The interfaces between the EVA layer and associated glass tablets are tied together through Tie constraints in ABAQUS.

As shown in Supplementary Figure 7, material properties measured from experiments are fed into our model. The stiff glass with the failure strength of 150 MPa, isotropic bulk modulus of 75 GPa, Poison ratio of 0.2 and density of 2.23 g/cm^3 were simulated using brittle cracking model in ABAQUS.

The soft EVA layers with the failure strength of 1.75 MPa, and critical energy release rate of 2 N/mm were modeled by cohesive elements with a trapezoidal cohesive law, which represents ideal elastic-plastic nonlinear behaviors due to tablets sliding and EVA elongation (Supplementary Figure 7b), and details of a typical cohesive zone model with trapezoidal cohesive law are shown in Supplementary Figure 7c. The quadratic form stress was adopted for damage initiation criterion:

$$\left(\frac{\langle\sigma\rangle}{\sigma_0}\right)^2+\left(\frac{\tau}{\tau_f}\right)^2=1 \tag{1}$$

where < > is the Macaulay bracket, σ and τ are tensile stress and shear stress, and we assumed the mixed-mode behavior was model-independent, thus the interfacial tensile strength (σ_0) and shear strength (τ_f) are taken as 1.75 MPa. After the damage initiation, the cohesive traction remains constant at maximum stress between certain separations followed by softening and failure. The trapezoidal shaped damage evolution was defined by tabular softening in ABAQUS. The softening response of cohesive element is defined as follows：

$$T_i=(1-D)K_i\delta_i \tag{2}$$

where D is scalar stiffness degradation (SDEG in ABAQUS), D = 0 represents that interface is undamaged, and D = 1 represents the interface is fully fractured. For the trapezoidal cohesive law, the damage evolution variable D can be directly defined as a tabular function of the effective displacement as follow:

$$D=\begin{cases}0, & 0\le\delta\le\delta_1\\ 1\text{-}\dfrac{\delta_1}{\delta}, & \delta_1\le\delta\le\delta_2\\ 1-\dfrac{\delta_1}{\delta}\dfrac{\delta_3-\delta}{\delta_3-\delta_2}, & \delta_2\le\delta\le\delta_3\\ 1, & \delta\ge\delta_3\end{cases} \tag{3}$$

where δ is separation displacement and details for δ_1 to δ_3 of nacre can be seen in **Supplementary Table 1**.

To simulate the impact responses of nacre-like structures and layered structures under different impact velocities, the models are simply supported by a rigid frame, and a cylindrical impactor with 0.75 kg in mass is treated as a rigid body and is fully constrained except in the loading direction. As shown in Supplementary Fig. 8, the failure morphologies and force-displacement curves obtained from

simulations are in good agreement with experimental results, which implies that our finite element model can well reveal the dynamics of energy dissipation in the laminated and nacre-like design under different impact velocities.

Supplementary Discussion 5: Effects of the aspect ratio of tablets and the layer thickness.

The glass sheet was engraved into 1600 Voronoi polygonal tablets and 225 Voronoi polygonal tablets to fabricate nacre-like structures with brick aspect ratios of 7.5 and 20 respectively. The experimental results show that with the increase in brick aspect ratios, the nacre-like structures exhibit the lower out-of-plane deformation and the critical impact velocity decreases (Supplementary Fig.10). The simulated force-displacement curves and energy dissipation - impact velocity curves for nacre-like structures with different aspect ratios are in good agreement with experiments (Supplementary Fig.11a-b). As the aspect ratio increases, the deformations of hard phase become concentrated underneath the impactor, the soft interlayer sliding is localized at the edges of tablets and the proportion of tablet-sliding induced failure area (A_{SF}/A_0) in each interlayer decreases (Supplementary Fig.11c-d). Further, we use the nonlinear shear-lag model to analyze the effects of aspect ratio on the interlayer load transfer (Fig. 5e), and the details of model can be seen in our previous works[3]. By solving the governing equation ($\partial\sigma/\partial x+\partial\tau/\partial y=0$) of representative volume element of nacre-like structure, we can get the distribution of interfacial shear stress,

$$\frac{\tau}{\tau_f}=\frac{2k^2}{\alpha^2}\left[c_5\sinh\left(2k\bar{x}\right)+c_6\cosh\left(2k\bar{x}\right)\right] \quad (4)$$

where $\bar{x}=\frac{x}{l_b}$, $\alpha=\sqrt{\frac{\tau_f l_b}{E_b t}}$, $k=\frac{l_b}{2l_c}$, $l_c=0.5t\sqrt{\frac{E_b}{G_m}\frac{1-\phi}{\phi}}$, l_b, t, E_b, G_m, ϕ are the brick length, the brick thickness, the elastic modulus of brick, the shear modulus of interface and the volume fraction of bricks, respectively. c_5, c_6 are the dimensional coefficients provided in our previous works[3]. Theoretical analysis shows that the distribution of interfacial shear stress becomes highly localized with the aspect ratio increase, which leads to the interlayer sliding localized at the edges of tablets, the energy dissipation decreases and thus the critical impact velocity decreases.

Meanwhile, by combing experiments, finite element simulations and theoretical analysis, we investigated the effects of glass-layer thickness on the impact performances of nacre-like structures and laminated structures. Experimental force F-displacement U curves in Supplementary Fig. 12a

show that for the structures with smaller glass-layer thickness, the reaction forces become higher and the out-of-plane deformations become smaller, which is consistent with previous findings that the fracture strength of composite increases with the layer thickness [4, 5]. Experimental energy dissipation E_{dis} - impact velocities V_{ini} curves in Supplementary Fig. 12b show that there always exists a critical impact velocity above which the laminated structures exhibit higher energy-dissipation compared to nacre-like structures, and the critical impact velocity increases significantly with decreasing glass-layer thickness. This trend originates from that the fracture strength of laminated structures significantly increases with the decrease of glass-layer thickness [4, 5], thus the initiation of high energy dissipation modes such as crack propagation in laminated structures with smaller t requires higher impact velocity. Further, we performed detailed investigations on the roles of glass-layer thickness t to the impact performances of nacre-like structures and laminated structures. As shown in Supplementary Fig. 13a, the laminated structures with smaller t dissipate lower energy at the range of low impact velocity, while the energy dissipations increase with the decrease of t when the impact velocity exceeds a critical value. In Supplementary Fig. 13b, the damaged area A_{dam}-V_{ini} curves for the laminated structures with different t show similar trends to the E_{dis} - V_{ini} curves under different t. Meanwhile, as shown in Supplementary Fig. 13c, we counted the number of cracks in the laminated structures with different t by selecting four centrosymmetric areas of sample and focusing the microscope field at different heights. As shown in Supplementary Fig. 13d-e, the laminated structures with smaller t show fewer cracks in each layer at the impact velocity of 2.0 m/s, while at the impact velocity of 5.0 m/s, more cracks occur in the laminated structures with thinner glass layer. This trend of crack numbers changing with t can be used to explain the energy-dissipation of the laminated structures with different t under different V_{ini}. Previous studies have shown that the fracture strength σ_{fr}^{lam} for the laminated structures can be described by the following equation [4]:

$$\frac{\sigma_{fr}^{lam}}{\sigma_{fr}^{\hom}} = \left(1-\kappa_E\right)\sqrt{\frac{2a_n}{\psi}\frac{R_p}{R_g}\frac{E_g}{E_p}}\sqrt{\frac{1}{t}} \tag{5}$$

From the Eq. 5, we can find that the σ_{fr}^{lam} of a crack in laminated structure also significantly increases with the decrease of t, thus the initiation of energy dissipation in laminated structures with smaller t requires higher impact velocity, and the energy dissipation of laminated samples with smaller t significantly increases in the range of high impact velocity. Therefore, at the range of low impact

velocity, fewer cracks occur in the laminated structures with smaller t and higher σ_{fr}^{lam} (Supplementary Fig. 13d), which leads to lower impact-energy dissipation. When the impact velocity exceeds a critical value, the perforation occurs in laminated structures, and more cracks occur in glass layers with smaller thicknesses (Supplementary Fig. 13e). Meanwhile, there are more glass layers in laminated samples with smaller t, thus the overall energy dissipation of laminated samples with smaller t is higher in the range of high impact velocity. In addition, based on above nonlinear shear-lag model (Supplementary Fig. 14a and Eq. 4), Supplementary Fig. 14b shows that at a fixed aspect ratio of l_b/t = 15 that is consistent with natural nacre[6-8], the distributions of interfacial shear stress are identical under different t (from t = 0.2 mm to t = 200 nm). And simulated results show that the proportion of tablet-sliding induced failure area (A_{SF}/A_0) in each interlayer of nacre-like structures remains constant under different t (Supplementary Fig. 14c). These phenomena indicate that at a fixed tablet aspect ratio, the layer thickness doesn't affect the distribution of tablet sliding, but only improves the overall energy dissipation of nacre-like structures by increasing the number of interlayers as t decreases within the same sample thickness. Based on the above analysis and Supplementary Fig. 14d, we can explain why the critical impact velocity V_{cri} significantly increases as the t decreases in experiments and simulations (Fig. 5e). As shown in Fig. 5e, with the decrease of layer-thickness t, the critical velocity V_{cri} increases significantly and satisfies a fitted scaling law of $V_{cri} \sim t^{-1/2}$. This indicates that the critical velocity can become tens of m/s when the layer thickness of the nacre-like structure decreases to nanoscale, which could explain why the nacre with nanoscale layer thickness couldn't keep superior impact resistance when the impact velocity is above ranging from 14.7 to 23.5 m/s in nature[7-11].

Supplementary Discussion 6: Effects of the sample thickness and the layer number.

Further, we performed investigations about the effects of sample thickness T and number of layers n on the critical impact velocity (Supplementary Fig.15a). As shown in Supplementary Fig. 15b-c, we investigated the force F-displacement U responses of the nacre-like structures, laminated structures and monolithic structures with different T under the impact velocity of 4 m/s, where the glass-layer thickness remains t = 0.2 mm and n varies with T. The energy dissipation E_{dis} - impact velocity V_{ini} curves in Supplementary Fig. 15d show that there is still a critical impact velocity as T increases. The measured values of energy dissipations for the samples with large thicknesses (T= 2.9 mm, n = 10) in this study are in good agreement with previous works[4]. Meanwhile, at the low impact velocity of V_{ini}

= 2.34 m/s, the nacre-like structures show higher energy dissipation than that of laminated structures and monolithic structures, which agrees well with the results in previous work[4]. However, the laminated structures exhibit higher energy dissipation than the nacre-like structures when the impact velocity exceeds a critical value.

Meanwhile, keeping the sample thickness as $T = 1.4$ mm, we analyzed the effects of the number of layers n on the impact performances of the nacre-like structures and laminated structures. Supplementary Fig. 16a shows the force F-displacement U responses of the samples with different n under the impact velocity of 4 m/s. Experimental E_{dis}-V_{ini} curves in Supplementary Fig. 16b show that there still exists the critical impact velocity, which increases significantly with n. Meanwhile, the simulated E_{dis} - V_{ini} curves in Supplementary Fig. 16c are in good agreement with experiments, showing that the critical impact velocity increases with n. Based on above fracture mechanics model (Eq. 5), we can find that the fracture strength σ_{fr}^{lam} of a crack in laminated structure with a fixed sample thickness significantly increases with the increase of n or the decrease of t, thus the initiation of energy dissipation in laminated structures with larger n requires higher impact velocity, and the energy dissipation of laminated samples with larger t significantly increases in the range of high impact velocity. Meanwhile, based on the analysis of above shear-lag model (Eq. 4 and Supplementary Fig. 14a), the distribution of tablet sliding in the interlayer of nacre-like structures mainly depend on the aspect ratio and not on the layer thickness or the layer number. Simulated results in Supplementary Fig. 16d show that when the aspect ratio and sample thickness are fixed, the proportion of tablet-sliding induced failure area (A_{SF}/A_0) in each interlayer of nacre-like structures remains nearly constant under different n. Therefore, the layer number doesn't affect the average area of tablets sliding in each interlayer, but only improves the overall energy dissipation of nacre-like structures with same sample thickness as the number of interlayers increases. Based on the above analysis and the schematic of the detailed analysis process (Supplementary Fig. 14d), we can explain why the critical impact velocity V_{cri} significantly increases as the n increases in experiments and simulations (Supplementary Fig. 16e). With the decrease of n, the V_{cri} increases significantly and satisfies a fitted scaling law of $V_{cri} \sim n^{1/2}$ (Supplementary Fig. 16e). This indicates that the critical velocity can become tens of m/s when the layer number of the nacre-like structure increases to hundreds/thousands, which could explain why the nacre with hundreds/thousands of layers couldn't keep superior impact resistance when the impact velocity is above ranging from 14.7 to 23.5 m/s in nature[7-11]. Although there are some differences

between the experimental settings and the natural impact process, above analysis indicates that as the number of layers increases, even for natural nacre with thousands of aragonite layers, there is always a critical velocity above which the nacre-like architecture is no longer a suitable design template for impact-resistant structures.

Supplementary Discussion 7: Effects of the contact radius and the bending length.

Further, we performed investigations about the effects of the impactor size R and the supporting plate (bending length L) on the finding of critical impact velocity (Supplementary Fig. 17a). Firstly, as shown in Supplementary Fig. 17b-c, we investigated the impact performances of the nacre-like structures, laminated structures and monolithic structures under different R, where L = 50 mm. Experimental force F-displacement U curves in Supplementary Fig. 17b show that the reaction forces and the out-of-plane deformations increase with the increase of R, and the monolithic structures and laminated structures both fail in a brittle fashion with high strength and low deformations, while the nacre-like structures exhibit more ductile responses with large deformations and low strength. The energy dissipation E_{dis} - impact velocity V_{ini} curves in Supplementary Fig. 17c show that there is still a critical impact velocity above which the laminated structures exhibit higher energy-dissipation compared to nacre-like structures, and the value of critical impact velocity increases with R. This trend originates from that when R is small, the deformations of all structures localize at the contact point, the out-of-plane deformation of sample is small and especially the energy-dissipations induced by tablet-sliding in nacre-like structures is suppressed (insets in Supplementary Fig. 17b). With the increase of R, the contact area significantly enlarges and the tablet-sliding in nacre-like structures and the failure pattern of radial and circumferential cracks in laminated structures become more pronounced over larger volumes.

Meanwhile, keeping the impactor size as R = 10 mm, we investigated the effects of the bending length L of the supporting plate on the impact performances of the nacre-like structures and laminated structures. Results in Supplementary Fig.18 show that there is always a critical impact velocity for different bending lengths L, while the bending length may influence the value of critical impact velocity, where the impactor size is R= 5 mm and the sample size is S= 60 mm. Even for impact tests under the plate support, our experimental results in Supplementary Fig. 19 show that there also exists the critical impact velocity. In addition, it should be remarked that in this work, we performed the impact tests on

the nacre-like structures, laminated structures and monolithic structures using a drop weight machine based on the guidelines given in the ASTM standard F3007[12]. In this work, the ratio between the bending length and side length of impacted samples is L/S= 83.3% (L = 50 mm, S = 60 mm), which is within the desirable ratio range of 80.6%-90.2% in the ASTM standard.

Supplementary Discussion 8: Drop tower testing for 3D printed samples.

To verify the generalizability of our conclusions, we further constructed 3D-printed nacre-like structures and laminated structures to analyze the influence of the impact velocity on the impact resistance of nacre-like structures. As shown in Supplementary Fig. 20a, a 3D printed nacre-like sample was constructed using an Object260 Connex3 3D printer (Statasys Ltd.). The 3D printed nacre-like structures are composed of five layers of 400-μm-thickness hard phases (VeroWhitePlus; Young's modulus is 0.8 ± 0.2 GPa) and four layers of 200-μm-thickness soft phases (TangoblackPlus; Young's modulus is 0.2 ± 0.05 MPa), the other geometry and arrangement are identical to that of the glass sample. The printer is capable to print multiple materials simultaneously and the excellent adhesion is achieved between stiff phases and soft phases due to the instantaneous in situ curing process. The characteristics of low-velocity impact resistance for the 3D-printed nacre-like structures were investigated using a drop tower testing machine with 1.55 kg hemispherical impactor. The impact conditions and data processing for the drop tower tests are same as that in method section.

As shown in Supplementary Fig. 20b, we investigated the force F-displacement U responses of the nacre-like structures and laminated structures under different impact velocities. The energy dissipation E_{dis} - impact velocity V_{ini} curves in Supplementary Fig. 20c demonstrate that nacre-like structures show superior energy-dissipation ability in the narrow range of low impact velocities, while they exhibit lower impact resistance than that of laminated structures when impact velocity exceeds a critical value, which is in good agreement with the testing results of synthetic bioinspired glass samples. Furthermore, Supplementary Fig. 20d shows the failure patterns at the back of the nacre-like structures and laminated structures under different V_{ini}. At a low V_{ini}, the "tablets sliding" in nacre-like architecture occurs easier and expands wider. By contrast, there are no visible cracks in the laminated structures at low V_{ini}, indicating low impact energy dissipation. However, when the V_{ini} exceeds the critical value, the tablet sliding slows down and even becomes localized at short time, while more cracks emerge and propagate in the laminated structures. Therefore, these results for 3D printed

samples further confirm that the anomalous inapplicability of nacre-like architecture as an impact-resistant template is not an occasional occurrence.

Supplementary Table 1. Material parameters of the trapezoidal cohesive law.

T_c	δ_1	δ_2	δ_3	G_c
1.75 MPa	0.5 mm	1.25 mm	1.5 mm	2 N/mm

Supplementary Figures

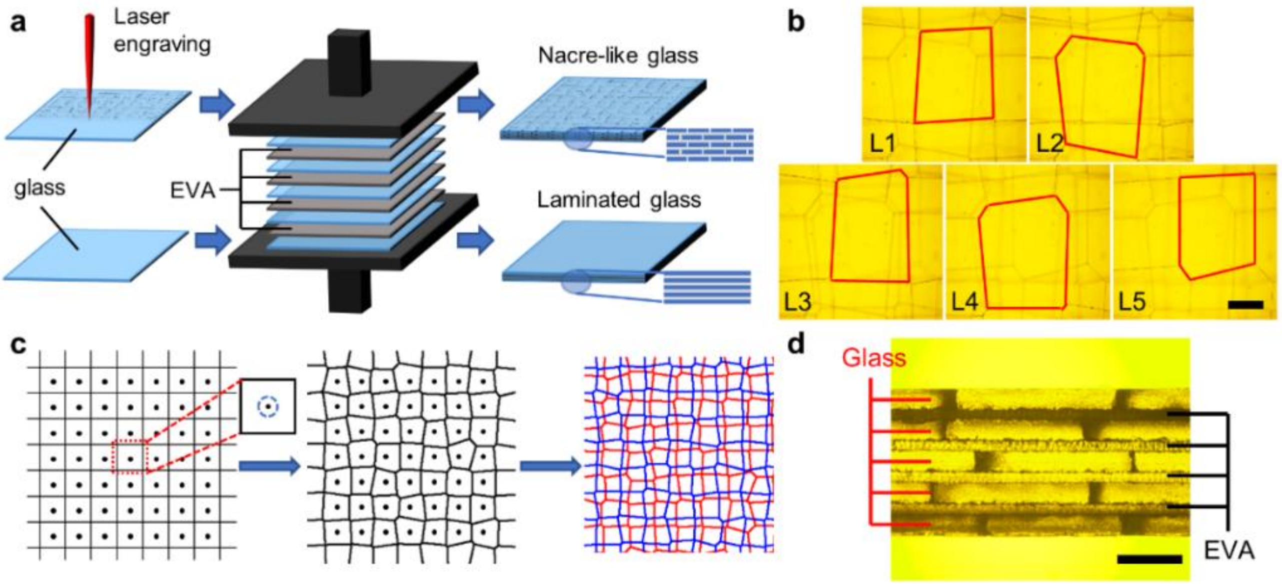

Supplementary Figure 1 | Preparation of the nacre-like structure plates. a, Manufacturing steps of the nacre-like glass and laminated glass. **b,** The laser-engraved pattern in each layer of the nacre-like glass. Scale bar, 250 μm. **c,** the Voronoi-shaped tables contours of the two layers. **d,** The cross-section of the nacre-like multilayered glass. Scale bar, 250 μm.

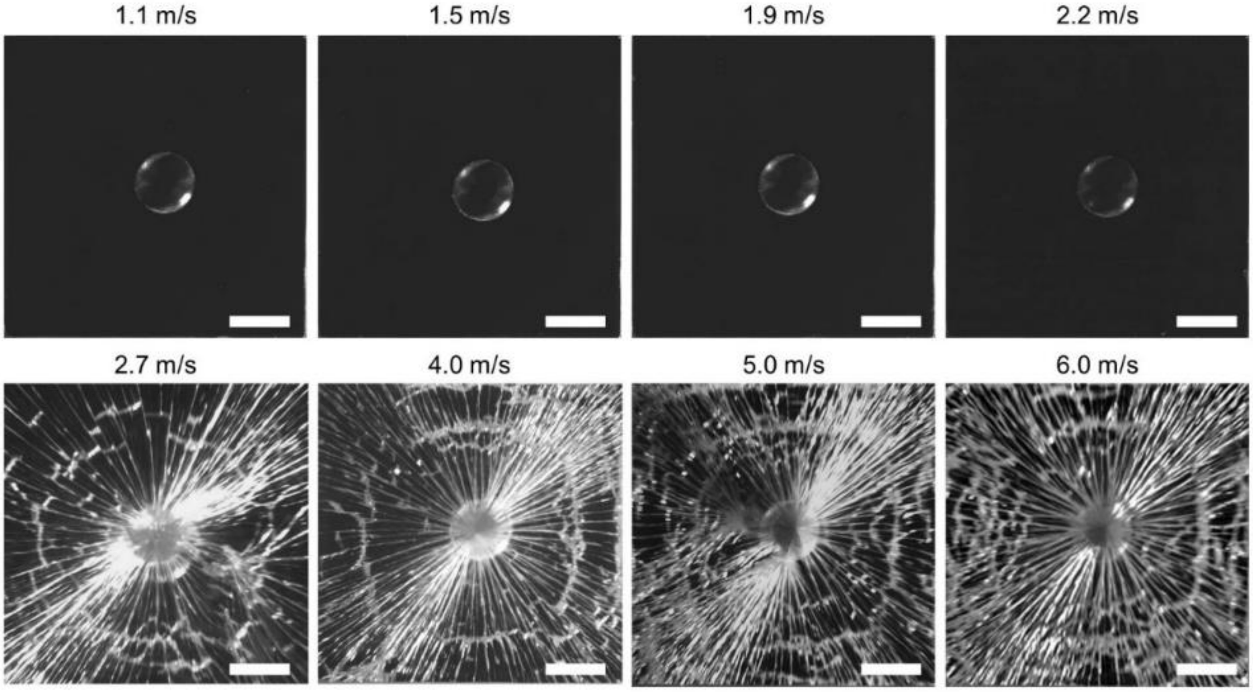

Supplementary Figure 2 | Damage morphology of the monolithic samples under different impact velocities. High-speed photography snapshots of the monolithic samples were taken from back of the impact faces, which show the damage patterns of samples. Scale bar: 10mm.

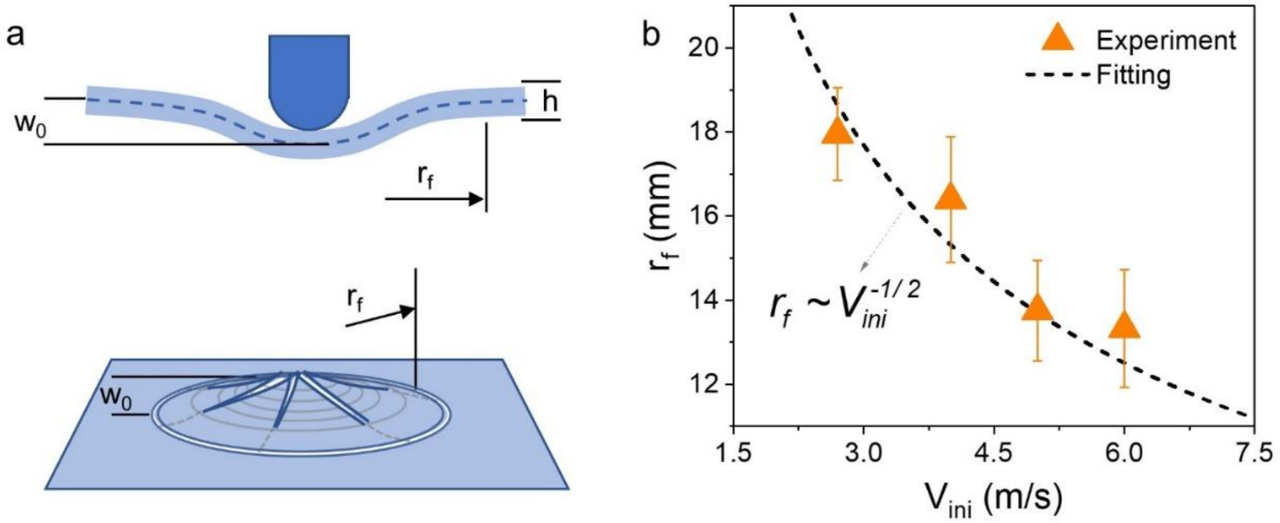

Supplementary Figure 3 | Crack pattern in the monolithic glass. a, The model charts the bending deformations and crack patterns in the glass plate under impact loads, where w_0 is the indentation, and r_f is the radius of the deformed region. **b,** Evolution of the r_f with impact velocity V_{ini}.

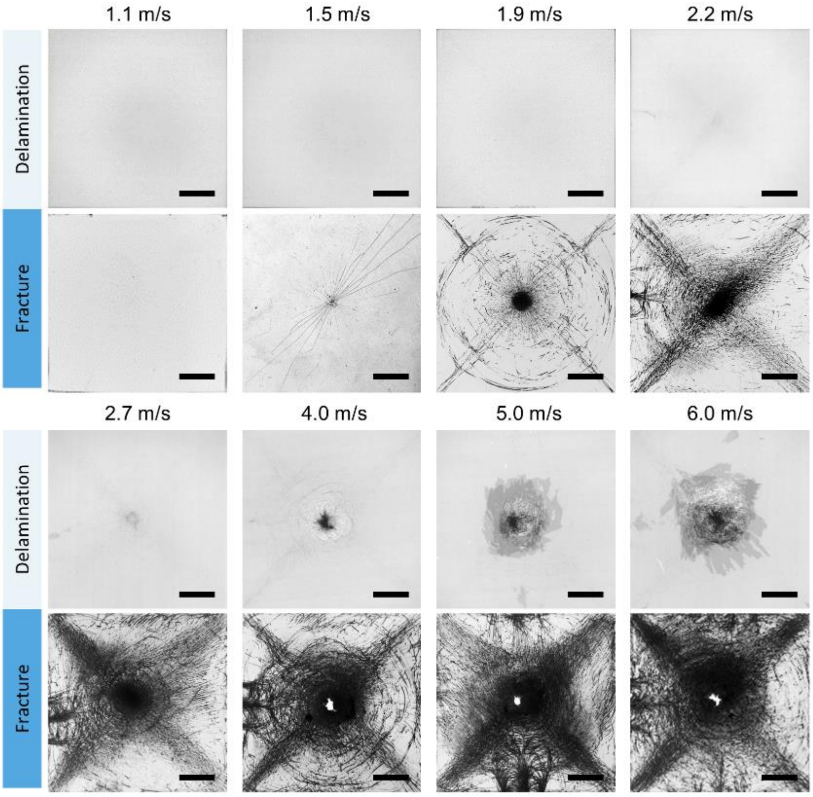

Supplementary Figure 4 | Damage morphology of the laminated samples under different impact velocities. The X-ray radiography images and photography images reflect the delamination and the glass fracture, respectively. Scale bar: 10mm.

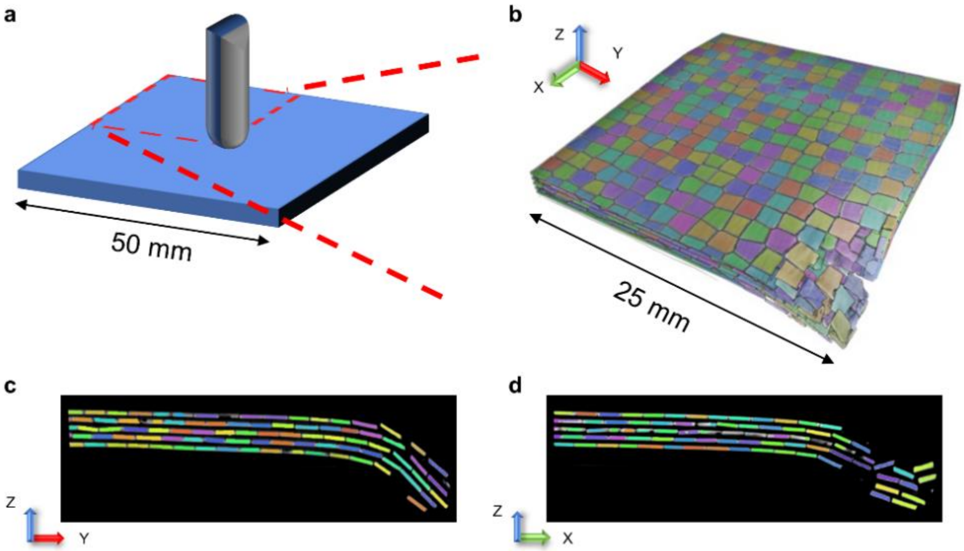

Supplementary Figure 5 | Micro-CT scan of the nacre-like sample punctured at 3.0 m/s impact velocity. a, The schematic of the scanning range, a quarter of the sample area selected. **b,** 3D microtomography of the sample, each colored tablet is reconstructed with area and position of the center. **c,** The cross-section of the sample in YZ-plane. **d,** The cross-section of the sample in XZ-plane.

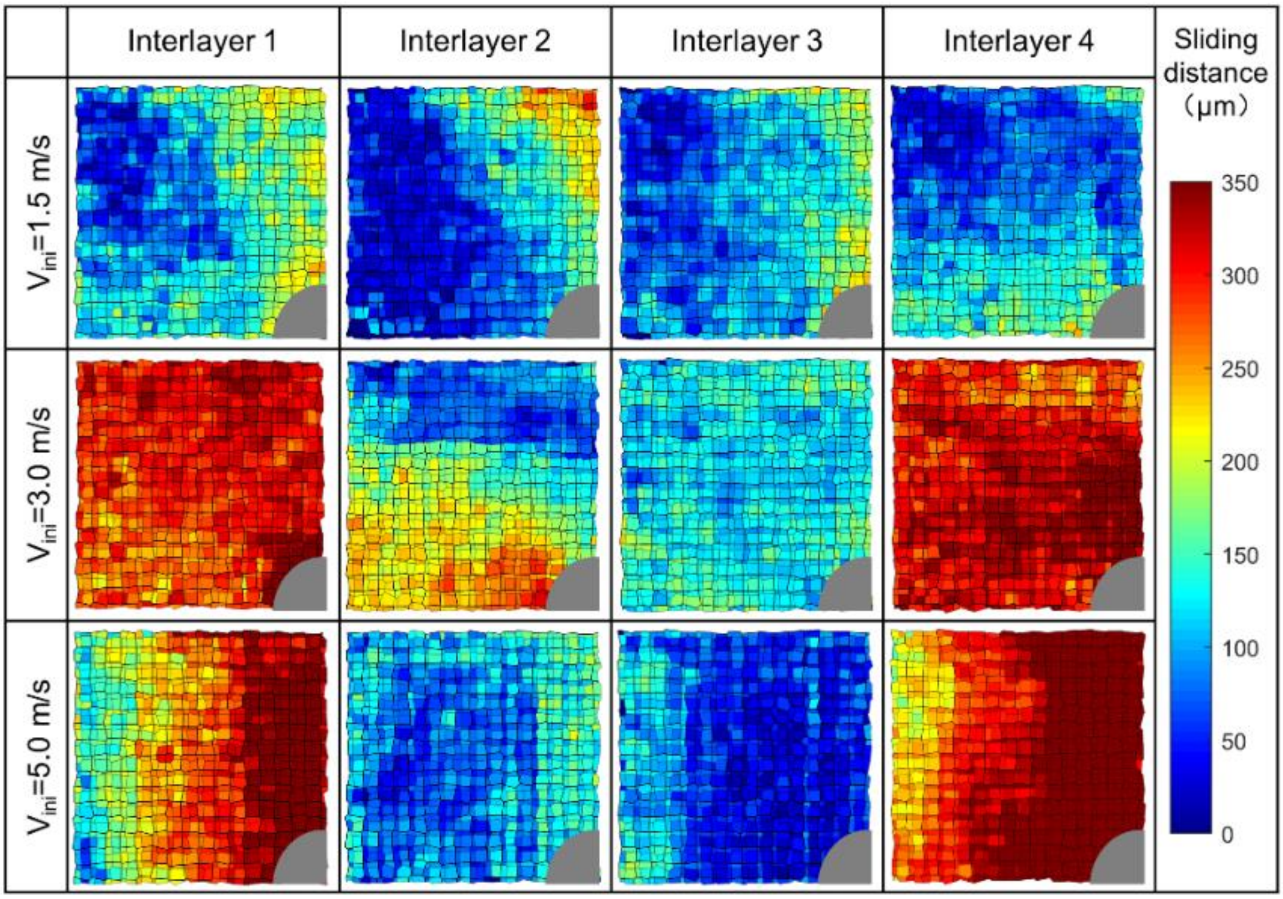

Supplementary Figure 6 | Map of sliding distances of each interlayer in the nacre-like samples under different impact velocities. A quarter of the sample area was selected for statistics, the gray fan in the lower right corner of these images represents the impactor head.

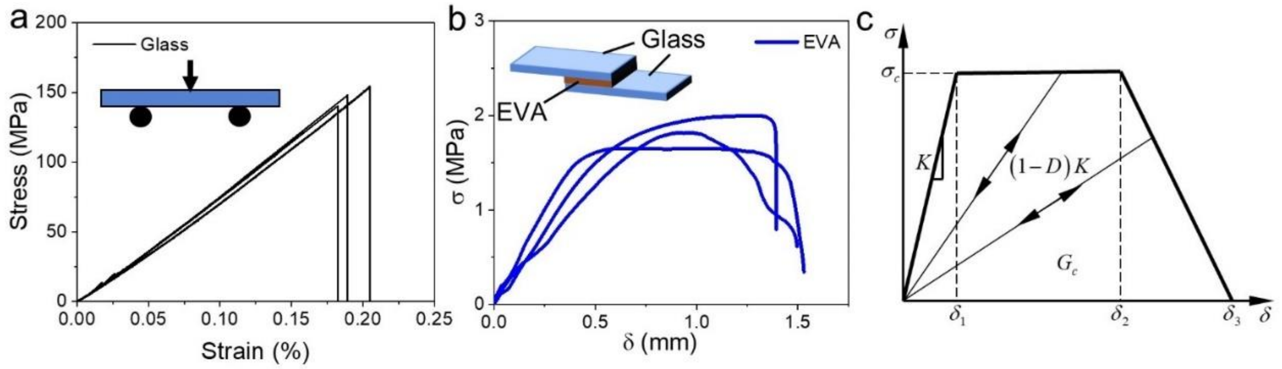

Supplementary Figure 7 | Properties of composite constituents. a, Three point bending tests on glass. **b,** Mode-II traction-displacement curves for single lap shear tests on interlayer EVA. **c,** Schematic cohesive zone model with a trapezoidal cohesive law.

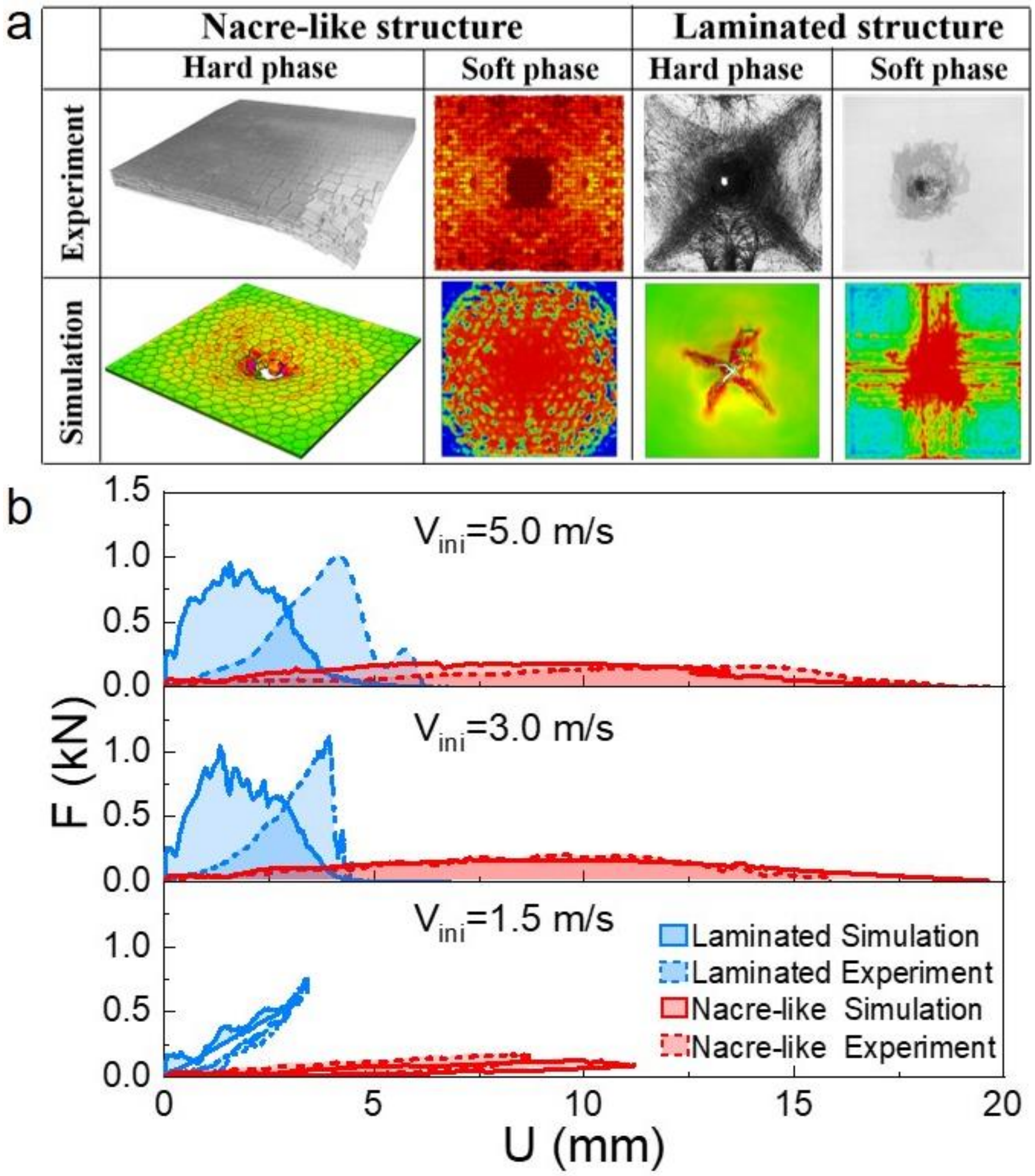

Supplementary Figure 8 | Comparisons between experiments and simulations. a, Comparisons of the failure morphologies. **b**, Experimental and simulated force F - displacement U curves for the nacre-like and laminated structures under different initial impact velocity V_{ini}.

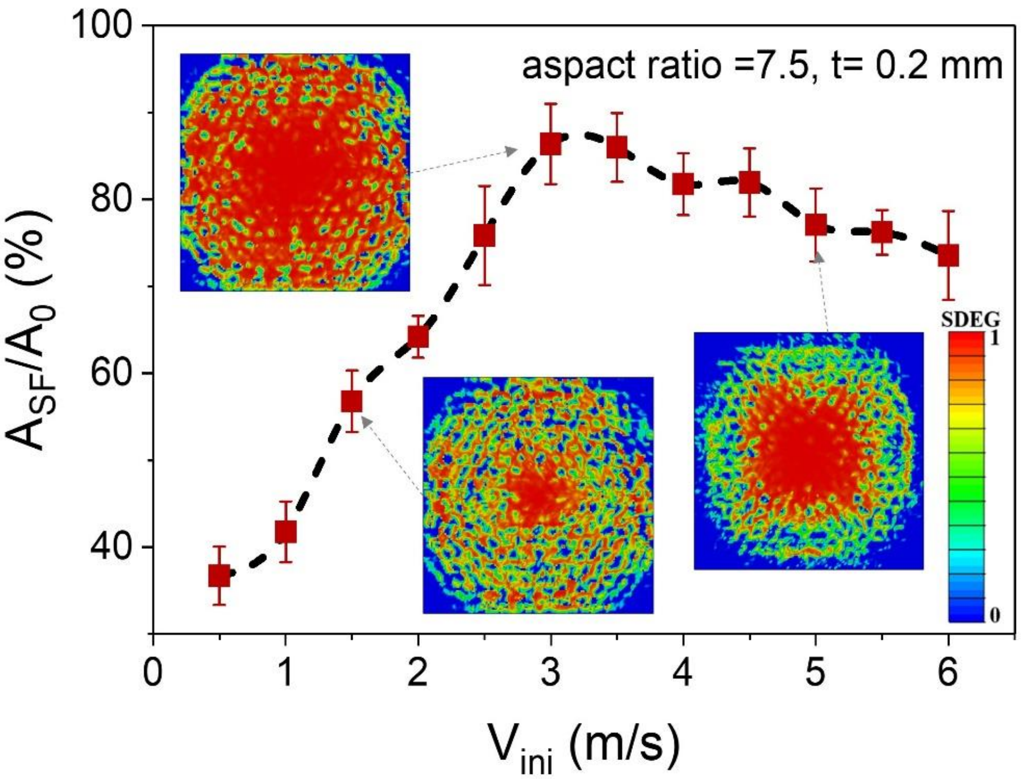

Supplementary Figure 9│The proportion of tablet-sliding induced failure area (A_{SF}/A_0) in each interlayer of nacre-like structures under different V_{ini}, where A_0 is the area of each interlayer. The insets show the distribution of tablet sliding in the lowermost interlayer, where SDEG = 0 represents zero failure while SDEG = 1 stands for the complete failure.

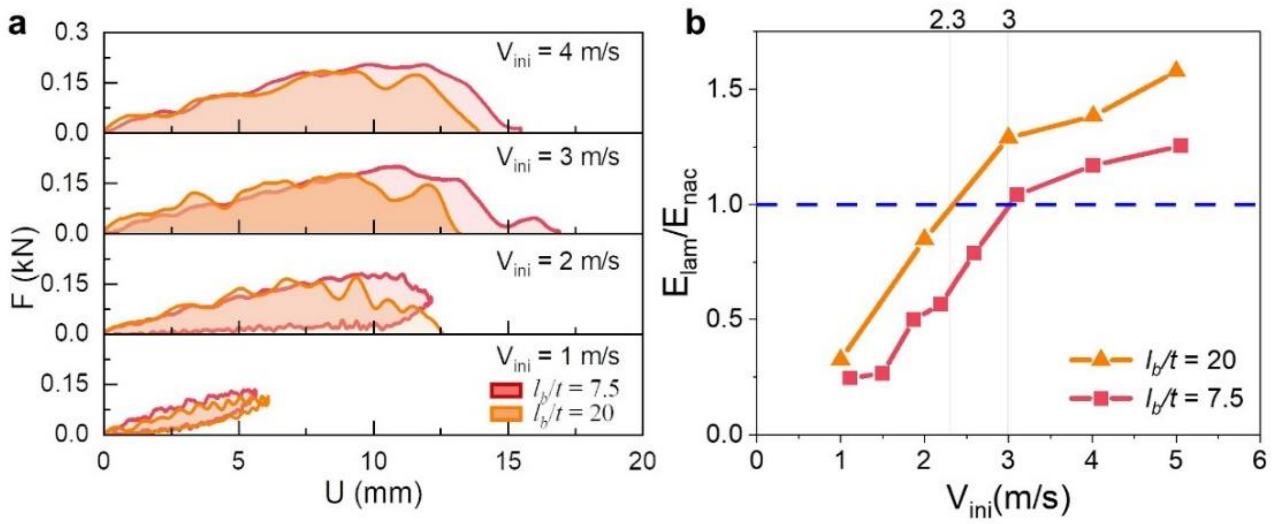

Supplementary Figure 10│Experiments for the effects of brick aspect ratios on the impact resistance of nacre-like structures. a, Experimental force F - displacement U curves of the nacre-like structures with different brick aspect ratios l_b/t. **b**, The ratio of energy dissipation of laminated structure E_{lam} to that of nacre-like structure E_{nac} with different l_b/t under different V_{ini}.

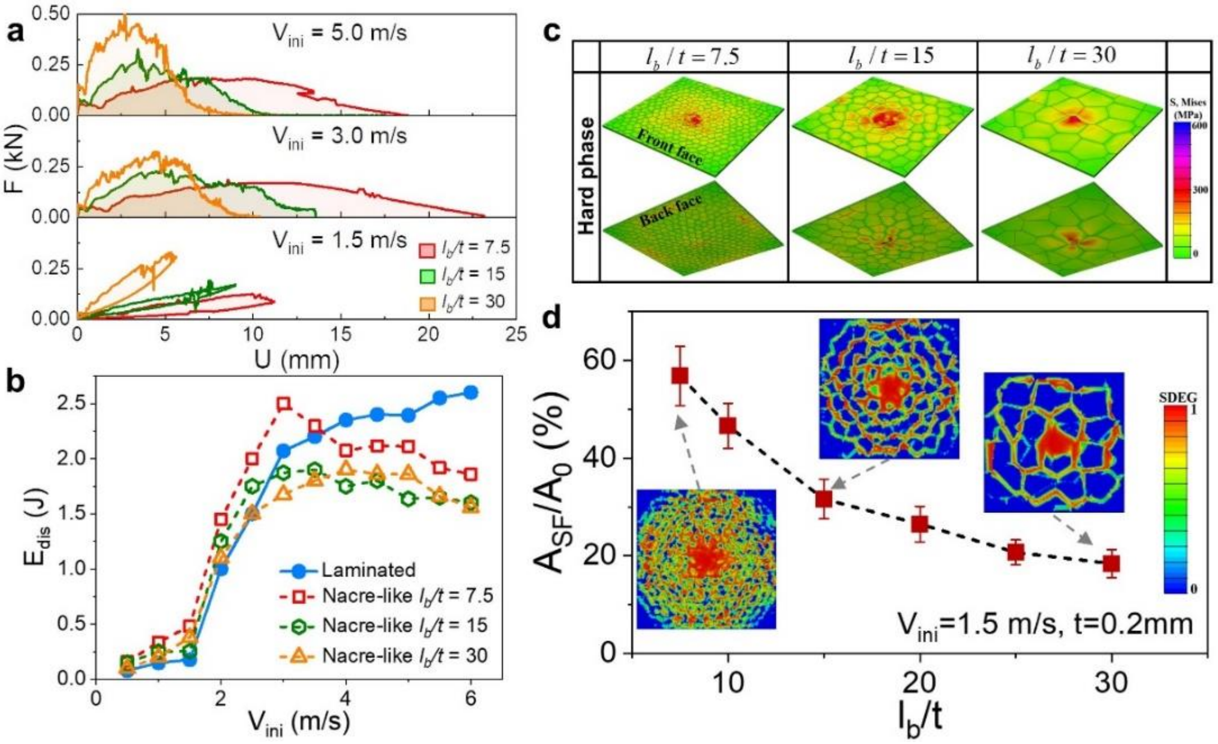

Supplementary Figure 11 | Simulations for the effects of brick aspect ratios on the impact resistance of nacre-like structures. a, Simulated force *F* - displacement *U* curves of the nacre-like structures with different brick aspect ratios l_b/t. **b,** Simulated energy dissipation E_{dis} - impact velocity V_{ini} for laminated samples and the nacre-like samples with different l_b/t. **c,** The deformation maps of the hard phase (von Mises stress field) in the nacre-like structures with different l_b/t under V_{ini} = 1.5 m/s. **d.** The proportion of tablet-sliding induced failure area (A_{SF}/A_0) in each interlayer of nacre-like structures with different l_b/t. The insets show the distribution of tablet sliding in the lowermost interlayer, where SDEG = 0 represents zero failure while SDEG = 1 stands for the complete failure.

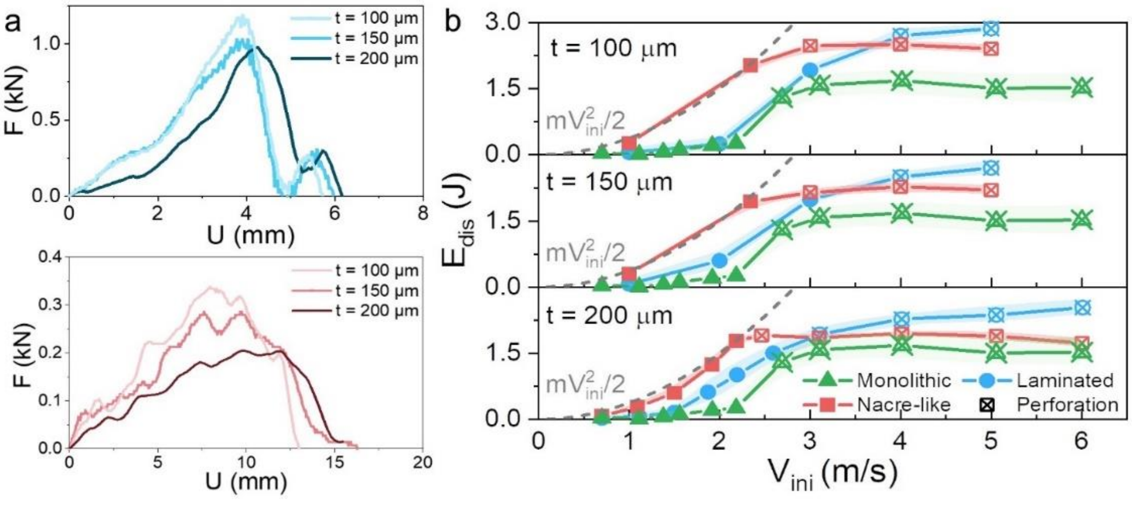

Supplementary Figure 12 | Effects of the layer thickness on the critical impact velocity. a, The

force *F*- displacement *U* curves for the laminated and nacre-like structures with different glass-layer thicknesses *t* under impact velocity of 4.0 m/s, where the size of samples and the volume fraction of soft phases remain constant. **b,** Experimental energy dissipation E_{dis} - impact velocities V_{ini} curves for the laminated and nacre-like structures with different *t*.

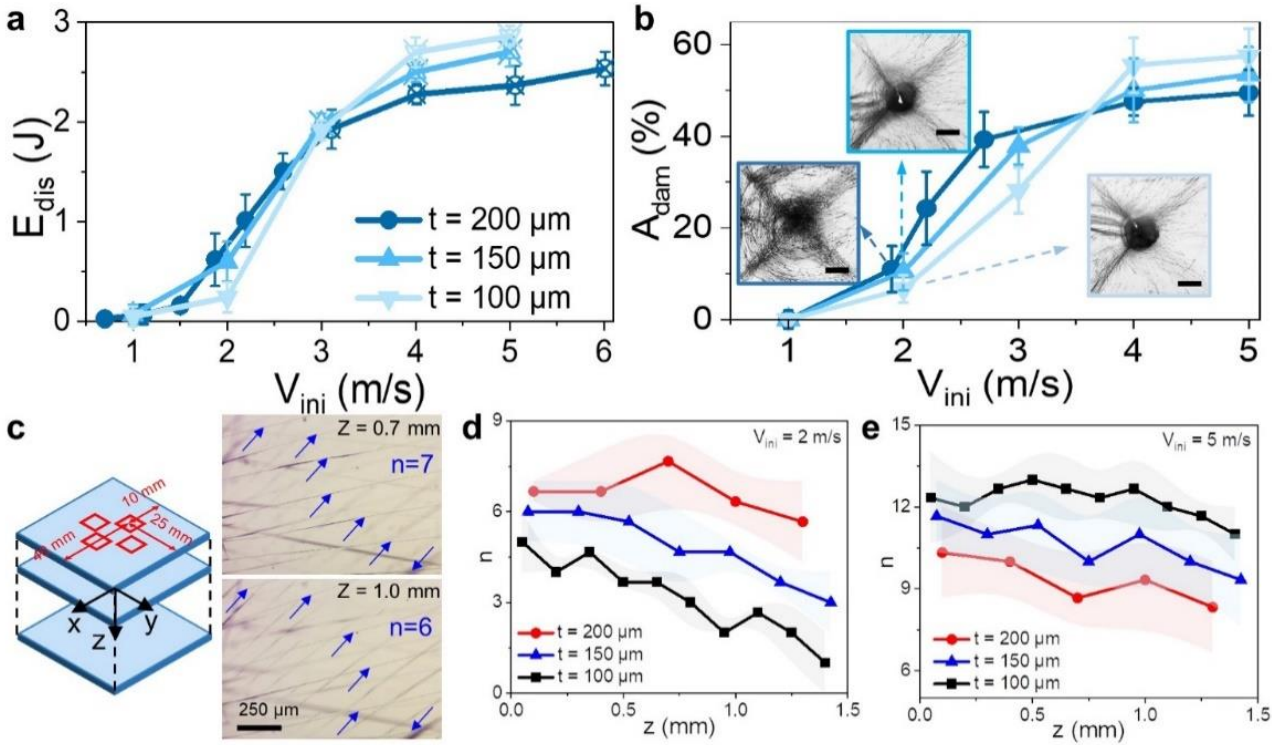

Supplementary Figure 13 | Effects of the layer thickness on the impact performances of laminated structures. a, The energy dissipation E_{dis} - impact velocities V_{ini} curves for the laminated structures with different glass thicknesses *t*. **b,** The proportion A_{dam} of damage area as functions of V_{ini} in the glass layers of laminated structures with different *t*, and the insets show crack morphologies under different *t*. Scale bar: 10 mm. **c,** The crack pattern in two adjacent 0.2 mm-thick glass layers of laminated structure under V_{ini} = 2.0 m/s. **d-e,** The number *n* of cracks along the thickness direction *z* in the laminated structures with different *t* at (d) V_{ini} = 2.0 m/s and (e) V_{ini} = 5.0 m/s. All the error bars represent the standard deviations of at least three replicate measurements.

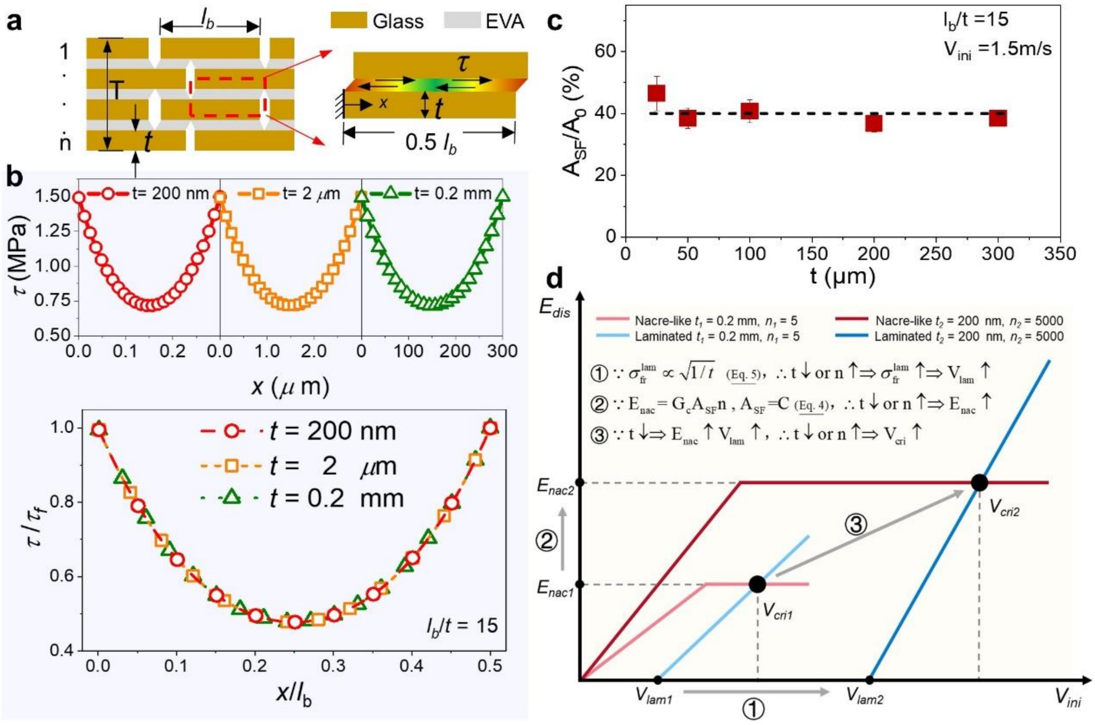

Supplementary Figure 14 | Analyses for the effects of the layer thickness. a, Schematics of the nonlinear shear-lag model. **b,** Analysis for shear stress distribution τ in the nacre-like structures with different *t* by the shear-lag model. **c,** The proportion of tablet-sliding induced failure area (A_{SF}/A_0) in each interlayer of nacre-like structures under different *t*, where A_0 is the area of each interlayer. **d,** Schematic for analyzing effects of the layer thickness *t* and the layer number *n*.

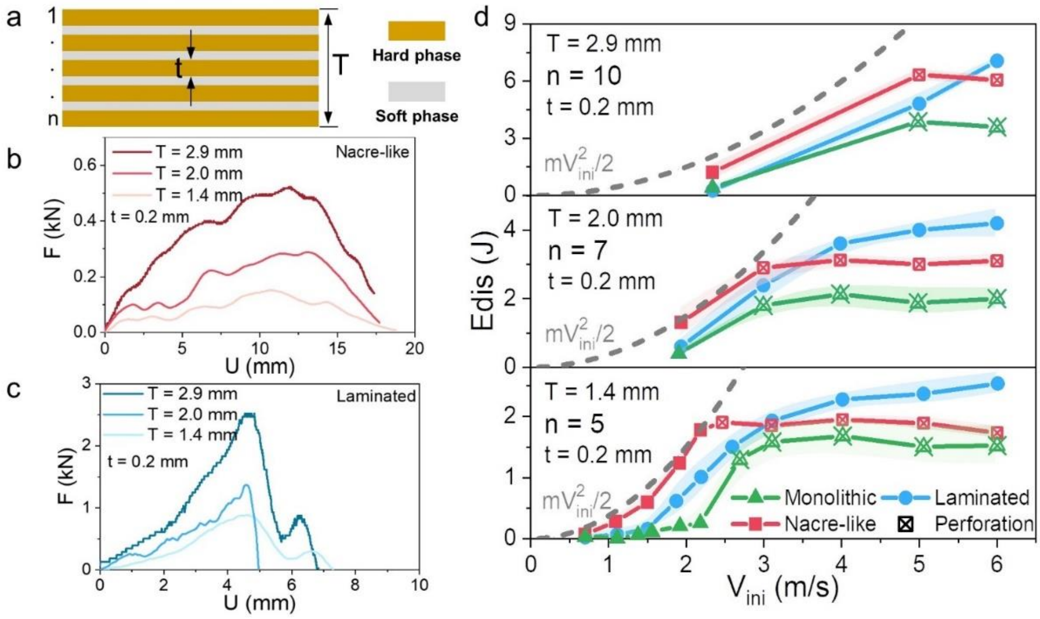

Supplementary Figure 15 | Effect of the sample thickness on the critical impact velocity. a, Schematic for the geometry of samples, including the sample thickness *T*, the layer thickness *t* and the

number of layers *n*. **b-c**, Force *F*-displacement *U* responses of the samples with different *T* under the impact velocity of 4 m/s, where the glass-layer thickness remains t = 0.2 mm and *n* varies with *T*. **d**, The energy dissipation E_{dis} - impact velocities V_{ini} curves for the samples with different *T* and *n*.

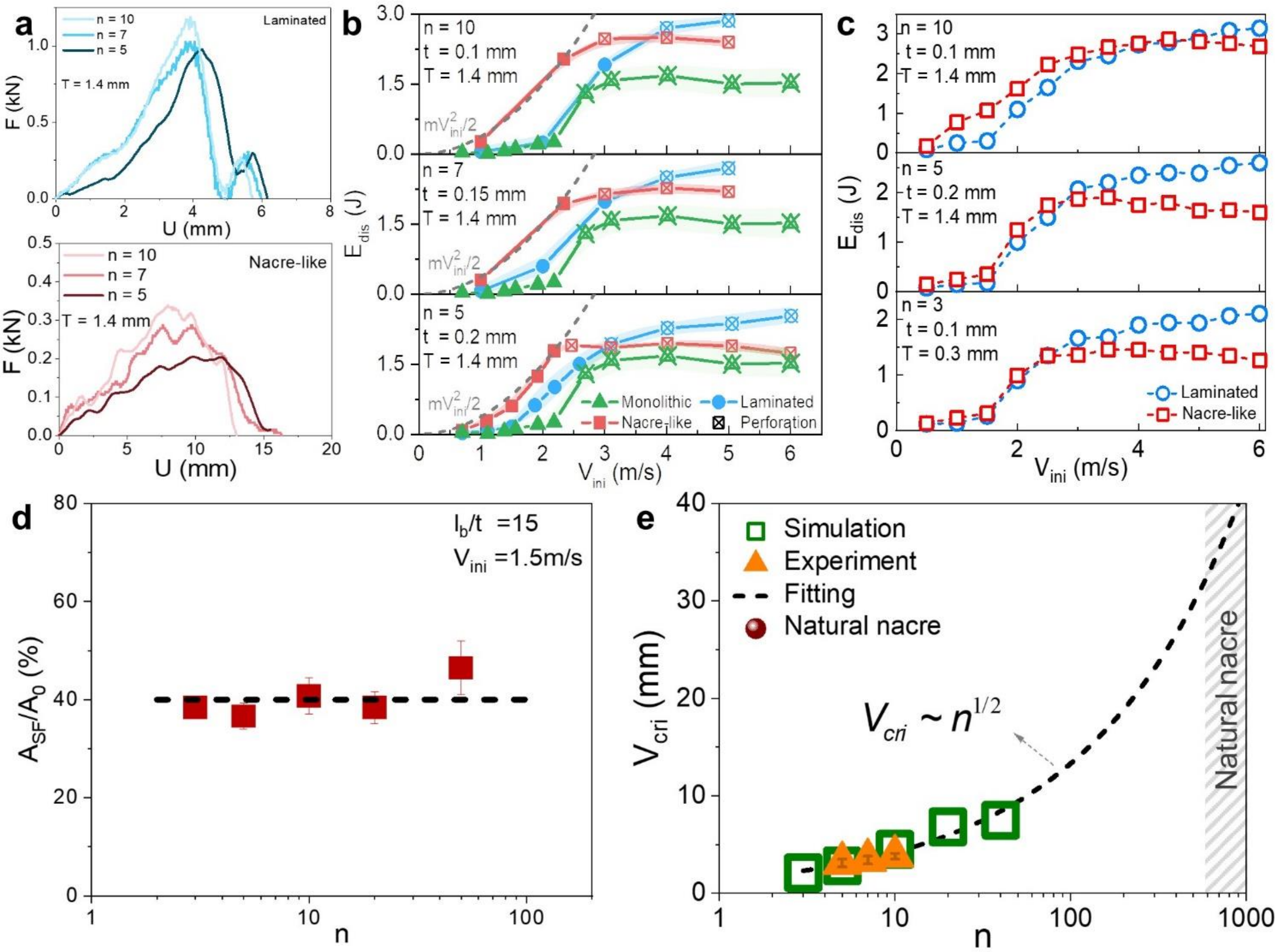

Supplementary Figure 16 | Effect of the number of glass layers on the critical impact velocity. a, Force *F*-displacement *U* responses for the samples with different *n* under the impact velocity of 4 m/s, where the sample thickness remains T = 1.4 mm and *t* varies with *n*. **b,** Experimental E_{dis} - V_{ini} curves for the samples with different *n*, where T = 1.4 mm and *t* varies with *n*. **c,** Simulated E_{dis} - V_{ini} curves for the samples with different *n*, where T = 1.4 mm and *t* varies with *n*. **d,** The proportion of tablet-sliding induced failure area (A_{SF}/A_0) in each interlayer of nacre-like structures with different *n*. **e,** The critical velocity V_{cri} as functions of *n*. All the error bars represent the standard deviations of at least three replicate measurements.

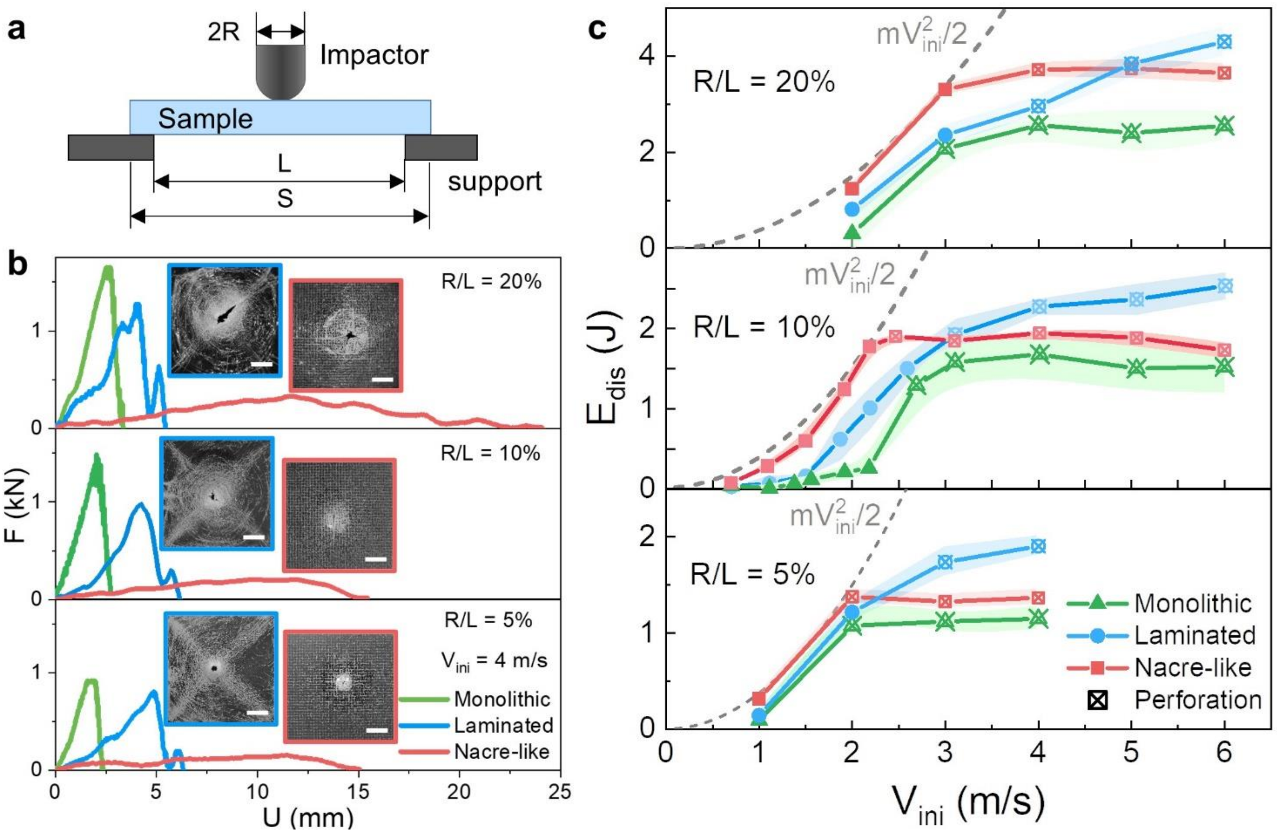

Supplementary Figure 17 | Effects of the contact radius on the critical impact velocity. a, Schematic for the impact tests under different impactor radius *R* and bending length *L*. **b**, The force *F* - displacement *U* curves for the monolithic samples, laminated samples, and nacre-like samples in the impact tests with different radius *R* of impactors, where *L* = 50 mm is the bending length. Scale bar: 10 mm. **c,** The energy dissipation E_{dis} - impact velocity V_{ini} in the impact tests with different *R*. All the error bars represent the standard deviations of at least three replicate measurements.

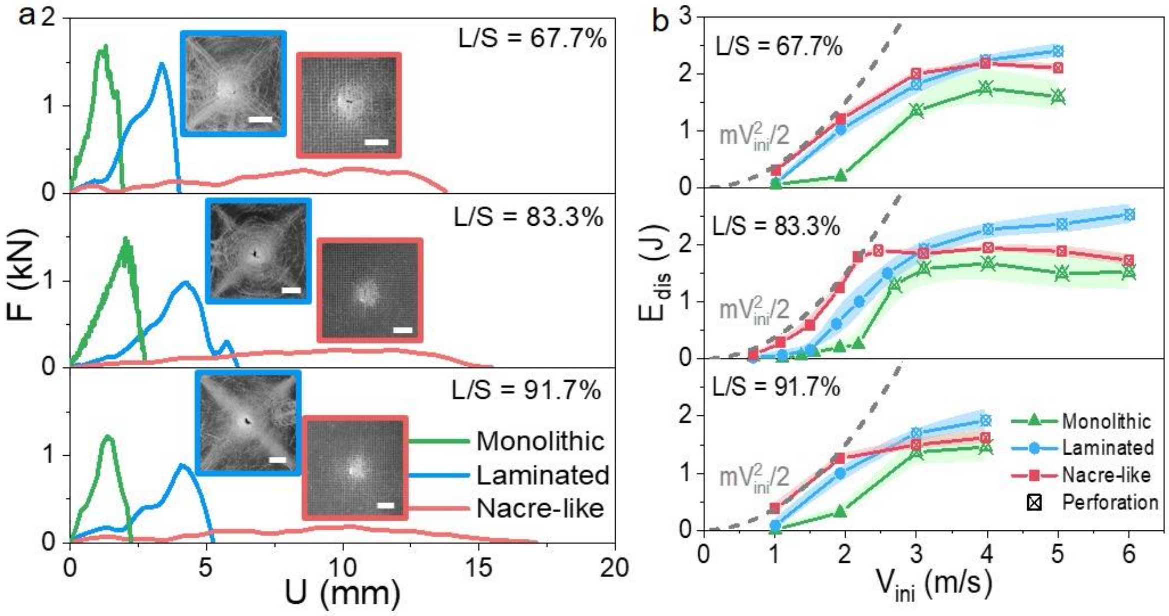

Supplementary Figure 18 | Effects of the bending length *L* on the critical impact velocity. a, The force *F* - displacement *U* curves for the monolithic samples, laminated samples, and nacre-like samples in the impact tests with different bending lengths *L*, where sample lengths *S* = 60 mm is the sample size. Scale bar: 10 mm. **b,** The energy dissipation E_{dis} - impact velocity V_{ini} in the impact tests with different *L*. All the error bars represent the standard deviations of at least three replicate measurements.

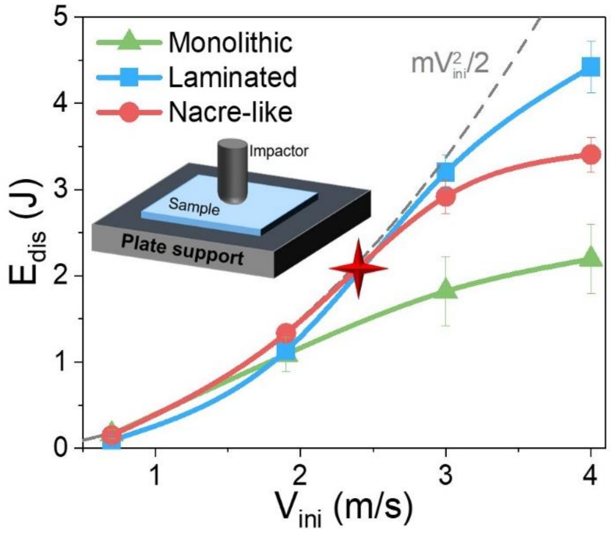

Supplementary Figure 19 | The energy dissipation E_{dis} - impact velocities V_{ini} curves for the laminated and nacre-like structures in the impact tests with plate support, where the cross symbol represents the critical impact velocity above which the nacre-like structures exhibit lower impact resistance than that of laminated structures. All the error bars represent the standard deviations of at least three replicate measurements.

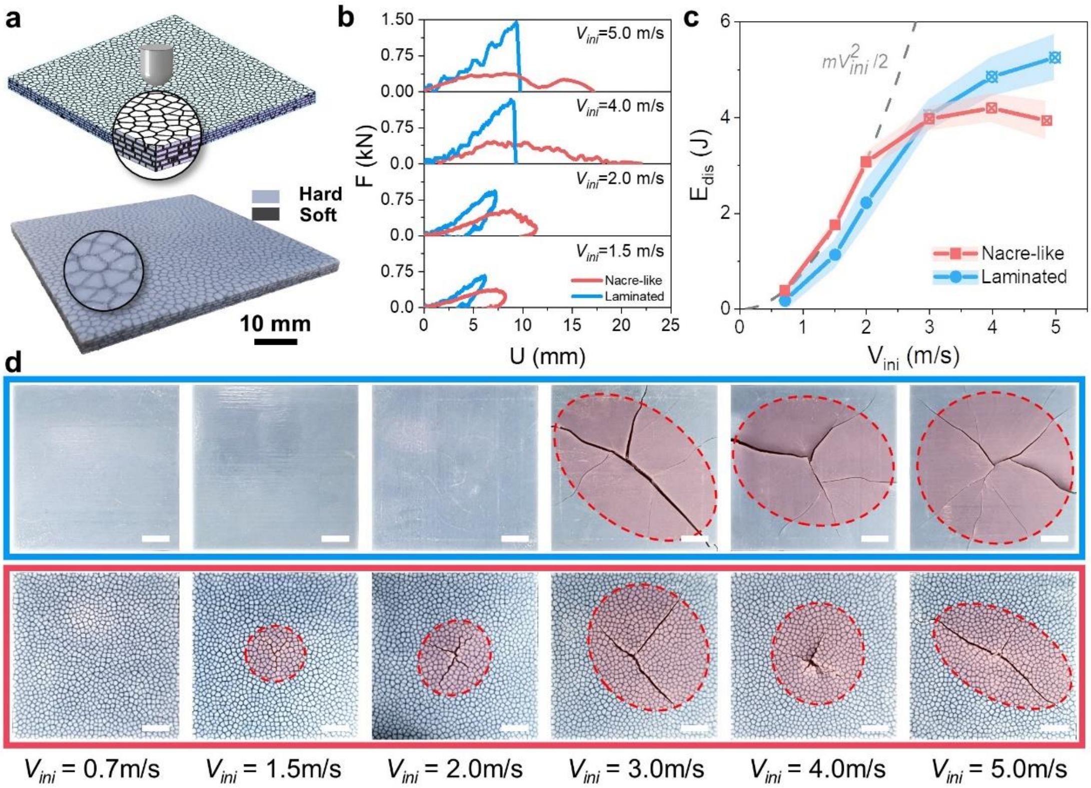

Supplementary Figure 20│Impact resistance of 3D printed samples under different impact velocities. a, 3D printing samples with nacre-like structure. **b**, The force *F*- displacement *U* curves for the laminated and nacre-like structures under different impact velocities. **c**, Experimental energy dissipation E_{dis} - impact velocities V_{ini} curves for the laminated and nacre-like structures. **d**, Damage patterns of 3D printing samples with laminated structures (top row) and nacre-like structures (bottom row) under different V_{ini}. Scale bar: 10 mm.

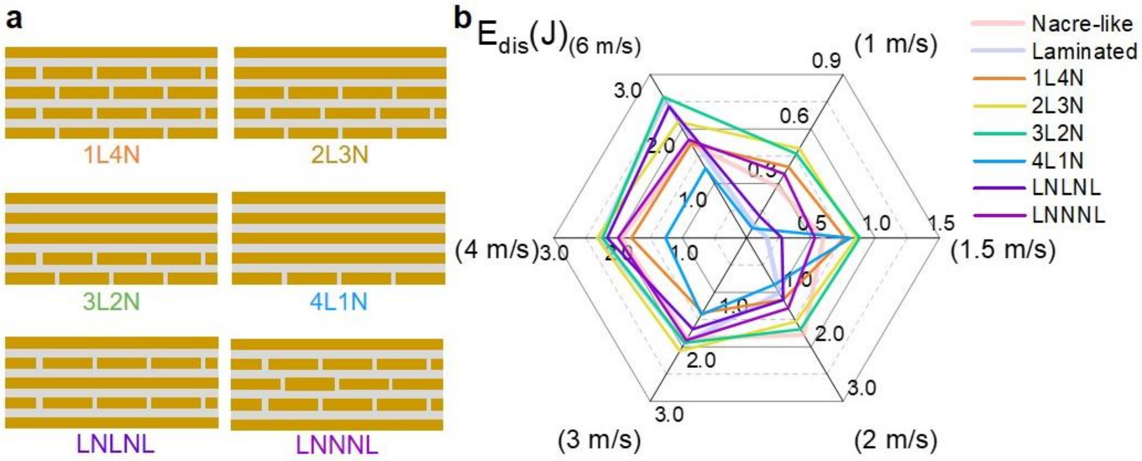

Supplementary Figure 21│Hybrid architectural panel design. a, Schematic of the 1L4N, 2L3N, 3L2N, 4L1N, LNLNL, LNNNL designs. **b,** Radar chart demonstrating the E_{dis} of samples under different impact velocities.

Supplementary References

1. Vandenberghe, N., Vermorel, R. & Villermaux, E. Star-shaped crack pattern of broken windows. *Physical Review Letters* **110**, 174302 (2013).
2. Chen, J., Xu, J., Liu, B., Yao, X. & Li, Y. Quantity effect of radial cracks on the cracking propagation behavior and the crack morphology. *Plos one* **9**, e98196 (2014).
3. Ni, Y., Song, Z., Jiang, H., Yu, S.-H. & He, L. Optimization design of strong and tough nacreous nanocomposites through tuning characteristic lengths. *Journal of the Mechanics and Physics of Solids* **81**, 41-57 (2015).
4. Kolednik, O., Predan, J., Fischer, F.D. & Fratzl, P. Bioinspired design criteria for damage-resistant materials with periodically varying microstructure. *Advanced Functional Materials* **21**, 3634-3641 (2011).
5. Liu, J., Zhu, W., Yu, Z. & Wei, X. Size effects in layered composites–Defect tolerance and strength optimization. *Composites Science and Technology* **165**, 154-160 (2018).
6. Yin, Z., Hannard, F. & Barthelat, F. Impact-resistant nacre-like transparent materials. *Science* **364**, 1260-1263 (2019).
7. Mayer, G. Rigid biological systems as models for synthetic composites. *Science* **310**, 1144-1147 (2005).
8. Barthelat, F., Tang, H., Zavattieri, P. & Li, C.-M., Espinosa H. On the mechanics of mother-of-pearl: a key feature in the material hierarchical structure. *Journal of the Mechanics and Physics of Solids* **55**, 306-337 (2007).
9. Li, X. et al. Spear and shield: Survival war between Mantis shrimps and abalones. *Advanced Materials Interfaces* **2**, 1500250 (2015).
10. Patek, S. N., Korff, W. L. & Caldwell, R. L. Deadly strike mechanism of a mantis shrimp. *Nature* **428**, 819-820 (2004).
11. Weaver, J.C. et al. The stomatopod dactyl club: a formidable damage-tolerant biological hammer. *Science* **336**, 1275-1280 (2012).
12. ASTM F3007-19. Standard Test Method for Ball Drop Impact Resistance of Laminated Architectural Flat Glass (Annual book of ASTM Standards, 2019).
